# The multifaceted role of mesenchymal stem cells in infectious diseases: antimicrobial properties, immunomodulation and tissue repair

**DOI:** 10.3389/fimmu.2026.1965154

**Published:** 2026-10-06

**Authors:** Omar Nyabi, Fadoua Temsamani, Assia Agalit, Karim El Bakkouri, Zahra Hendi, Feras Al Battah, Massoud Vosough, Karima Idrissi Serhrouchni, Mustapha Najimi

**Affiliations:** 1 Center for Applied Molecular Technologies, Institute of Clinical and Experimental Research, UCLouvain, Brussels, Belgium; 2 Laboratory of Histology, Embryology and Cytogenetics, Faculty of Medicine and Pharmacy of Tangier, Abdelmalek Essaâdi University, Tangier, Morocco; 3 Mohammed VI Center of Research and Innovation, Mohammed VI University of Sciences and Health, Rabat, Morocco; 4 Department of Regenerative Medicine, Cell Science Research Center, Royan Institute for Stem Cell Biology and Technology, Academic Center for Education, Culture and Research (ACECR), Tehran, Iran; 5 Department of Biology and Biotechnology, Arab American University, Jenin, Palestine; 6 Private University of Marrakech, Marrakech, Morocco; 7 Liver Therapy & Evolution Team, In Vitro Toxicology and Dermato-Cosmetology (IVTD), Faculty of Medicine and Pharmacy, Vrije Universiteit Brussel, Brussels, Belgium

**Keywords:** antimicrobial properties, cell therapy, immunomodulation, infectious diseases, mesenchymal stem cells, tissue regeneration

## Abstract

**Background:**

Mesenchymal stem cells (MSCs) have emerged as promising therapeutic agents for infectious diseases owing to their antimicrobial, immunomodulatory, and tissue regenerative properties. Beyond their well-established role in tissue repair, MSCs and their secretome directly influence pathogen clearance through the secretion of antimicrobial peptides, extracellular vesicles, iron-sequestration mechanisms, and modulation of both innate and adaptive immune responses.

**Knowledge gap:**

Despite extensive preclinical investigations, MSC responses are highly pathogen-dependent, with outcomes that can be beneficial, neutral, or even detrimental depending on the infectious context. The heterogeneity in MSC efficacy across different pathogens underscores the need for a nuanced understanding of their mechanisms of action and the development of strategies to optimize therapeutic outcomes.

**Key findings:**

In bacterial infections, MSCs generally promote pathogen clearance and attenuate inflammation. Conversely, antiviral effects are often indirect, primarily involving the regulation of cytokine-driven immunopathology rather than direct inhibition of viral replication. Fungal and parasitic infections exhibit more complex interactions, with some pathogens exploiting MSCs as protective niches or reprogramming their immunological functions. Emerging strategies, including priming, genetic engineering, and cell-free secretome approaches, show promise in enhancing MSC efficacy. However, heterogeneity in cell source, manufacturing processes, potency, dosing, and timing remains a significant translational barrier.

**Conclusion:**

Future development of MSC-based therapies should shift from a universal, one-size-fits-all approach toward pathogen-specific, tailored cellular or cell-free products. This transition requires the establishment of standardized potency assays, optimized dosing protocols, and clinically meaningful endpoints. Adopting a pathogen-oriented framework will be essential for advancing MSCs as effective anti-infective agents, especially in the face of rising antimicrobial resistance.

## 1 Introduction

Mesenchymal stem cells (MSCs) are multipotent stromal cells that can be isolated from a wide range of tissues, including bone marrow, adipose tissue, perinatal tissues, dental pulp, peripheral blood, lung, and heart (1–5). Beyond their differentiation potential, MSCs have attracted considerable attention because of their potent immunomodulatory, anti-inflammatory, anti-fibrotic, anti-apoptotic, and regenerative properties (6). These characteristics have driven extensive investigation of MSCs as therapeutic agents for a broad spectrum of disorders, including autoimmune, cardiovascular, neurological, and inflammatory diseases (7). Over the last decade, a growing body of evidence has revealed that MSCs possess additional functions extending beyond tissue regeneration. In particular, MSCs and their secretome exhibit direct and indirect antimicrobial activities mediated through antimicrobial peptides (AMPs), extracellular vesicles (EVs), cytokines, and modulation of innate and adaptive immune responses (8, 9). These properties have generated increasing interest in MSCs as potential adjunctive therapies for infectious diseases, especially in the context of rising antimicrobial resistance (AMR).

AMR represents one of the most pressing global health challenges of the twenty-first century. According to the Global Burden of Disease study, bacterial AMR was associated with nearly five million deaths worldwide in 2019 (10). In response, the World Health Organization has identified AMR as a major global priority and established a dedicated research agenda to accelerate the development of innovative therapeutic strategies (11). While conventional antimicrobial agents directly target pathogens, MSC-based therapies offer a fundamentally different approach by simultaneously enhancing pathogen clearance, modulating dysregulated immune responses, limiting tissue damage, and promoting tissue repair.

Encouraging preclinical and early clinical studies have demonstrated the therapeutic potential of MSCs in bacterial sepsis, pneumonia, acute respiratory distress syndrome (ARDS), chronic wounds, and several viral infections, including COVID-19 (12). Nevertheless, accumulating evidence indicates that MSC-mediated responses are highly context-dependent. While MSCs generally exhibit beneficial effects in bacterial infections, their efficacy in viral diseases remains variable, and their interactions with fungal and parasitic pathogens can produce both protective and detrimental outcomes. Such observations suggest that the antimicrobial functions of MSCs are not universal but are shaped by pathogen-specific and microenvironmental factors.

Several reviews have summarized the regenerative and immunomodulatory properties of MSCs in infectious diseases. However, most have focused on specific pathogens or individual disease categories. A comprehensive comparative analysis of MSC responses across bacterial, viral, fungal, and parasitic infections remains lacking. Understanding the common and divergent mechanisms governing these interactions is essential for identifying the most promising therapeutic indications and for overcoming current translational barriers.

In this review, we critically examine the antimicrobial, immunomodulatory, and regenerative functions of MSCs across the major classes of infectious diseases. Rather than providing a descriptive overview of individual studies, we focus on identifying pathogen-specific patterns, mechanistic similarities and differences, and factors underlying beneficial, neutral, or adverse outcomes. We further discuss emerging strategies to enhance MSC efficacy, including priming, genetic engineering, and cell-free approaches, and highlight the key challenges that must be addressed to enable the successful clinical translation of MSC-based anti-infective therapies.

This narrative review was conducted through targeted searches of PubMed, Scopus, and Web of Science, supplemented by ClinicalTrials.gov to identify relevant clinical studies. The search encompassed publications from approximately 2006 to August 2026. Emphasis was placed on peer-reviewed mechanistic, preclinical, and clinical investigations involving MSCs or MSC-derived products in the context of bacterial, viral, fungal, or parasitic infections. Evidence was categorized as preclinical when derived from *in vitro* experiments or animal models, and as clinical when obtained from human interventional studies, registered trials, or clearly documented clinical reports. Additionally, references were screened from pertinent review articles and primary studies to ensure comprehensive coverage of key mechanistic insights and translational developments.

## 2 Mechanisms of antimicrobial action of MSCS

### 2.1 Direct antimicrobial mechanisms

The antimicrobial activity of MSCs is mediated by a sophisticated secretome comprising AMPs, soluble factors, EVs, and metabolic regulators, collectively functioning to inhibit pathogen survival and dissemination (**Figure 1**). Rather than relying on a singular antimicrobial pathway, MSCs utilize a multifaceted array of mechanisms, including direct pathogen inhibition, nutrient deprivation, and modulation of host innate immune responses. Among these, the secretion of AMPs represents one of the most extensively characterized antimicrobial strategies. Notably, LL-37, β-defensin-2 (BD2), hepcidin (HAMP), and lipocalin-2 (LCN2) serve as principal antimicrobial effectors produced by MSCs (8, 13). LL-37, a member of the cathelicidin family, exhibits broad-spectrum antimicrobial activity against bacteria, fungi, and certain viruses through mechanisms such as disruption of microbial membranes, neutralization of endotoxins, and promotion of immune cell recruitment to sites of infection (14, 15). Beyond its antimicrobial activity, LL-37 plays a pivotal role in tissue repair and angiogenesis, underscoring the dual regenerative and antimicrobial functions of MSC-derived secreted factors (16). Similarly, β-defensin-2 (BD2) contributes to host defense not only through direct antimicrobial effects but also by modulating inflammatory responses. Its expression is upregulated by bacterial products and pro-inflammatory cytokines, thereby linking AMP production to the local infectious milieu (17). In experimental models of Escherichia coli pneumonia, MSC-derived BD2 enhanced bacterial clearance and mitigated inflammation via Toll-like receptor 4 (TLR4)-dependent mechanisms, exemplifying the intricate interplay between antimicrobial and immunomodulatory functions (18). A second principal antimicrobial strategy employed by MSCs involves nutrient restriction; specifically, HAMP and LCN2 limit microbial access to iron, an essential nutrient for pathogen proliferation. HAMP regulates systemic iron homeostasis by inducing ferroportin internalization and degradation, leading to a decreased extracellular iron availability (19, 20). LCN2 effectively sequesters bacterial siderophores, which limits iron availability to pathogens and impedes their proliferation (21). In models of bacterial pneumonia, MSC-derived LCN2 plays a pivotal role in pathogen clearance, acting through both direct antimicrobial mechanisms and indirect modulation of host immune responses via interactions with alveolar macrophages. This highlights the essential role of nutritional immunity in MSC-mediated antimicrobial defense (22). In addition to classical AMPs, MSCs produce a spectrum of other antimicrobial mediators, including cystatin C and elafin, which contribute to their antimicrobial repertoire (23). The dual roles of indoleamine 2,3-dioxygenase (IDO) as an antimicrobial and immunoregulatory agent are discussed in Section 2.2. Importantly, AMP expressions are not constitutive but are dynamically regulated by environmental stimuli, such as bacterial exposure, pathogen-associated molecular patterns, and inflammatory cytokines, resulting in the upregulation of LL-37, BD2, HAMP, and LCN2. This adaptive response suggests that MSCs modulate their antimicrobial arsenal in accordance with the infectious milieu, potentially underpinning the observed pathogen-dependent variability in therapeutic outcomes (8). Beyond soluble mediators, EVs, including exosomes and microvesicles, have emerged as critical mediators of MSC antimicrobial activity. These vesicles deliver a diverse cargo of proteins, cytokines, messenger RNAs, and microRNAs that influence pathogen clearance and shape host immune responses (24, 25).

**Figure 1:**
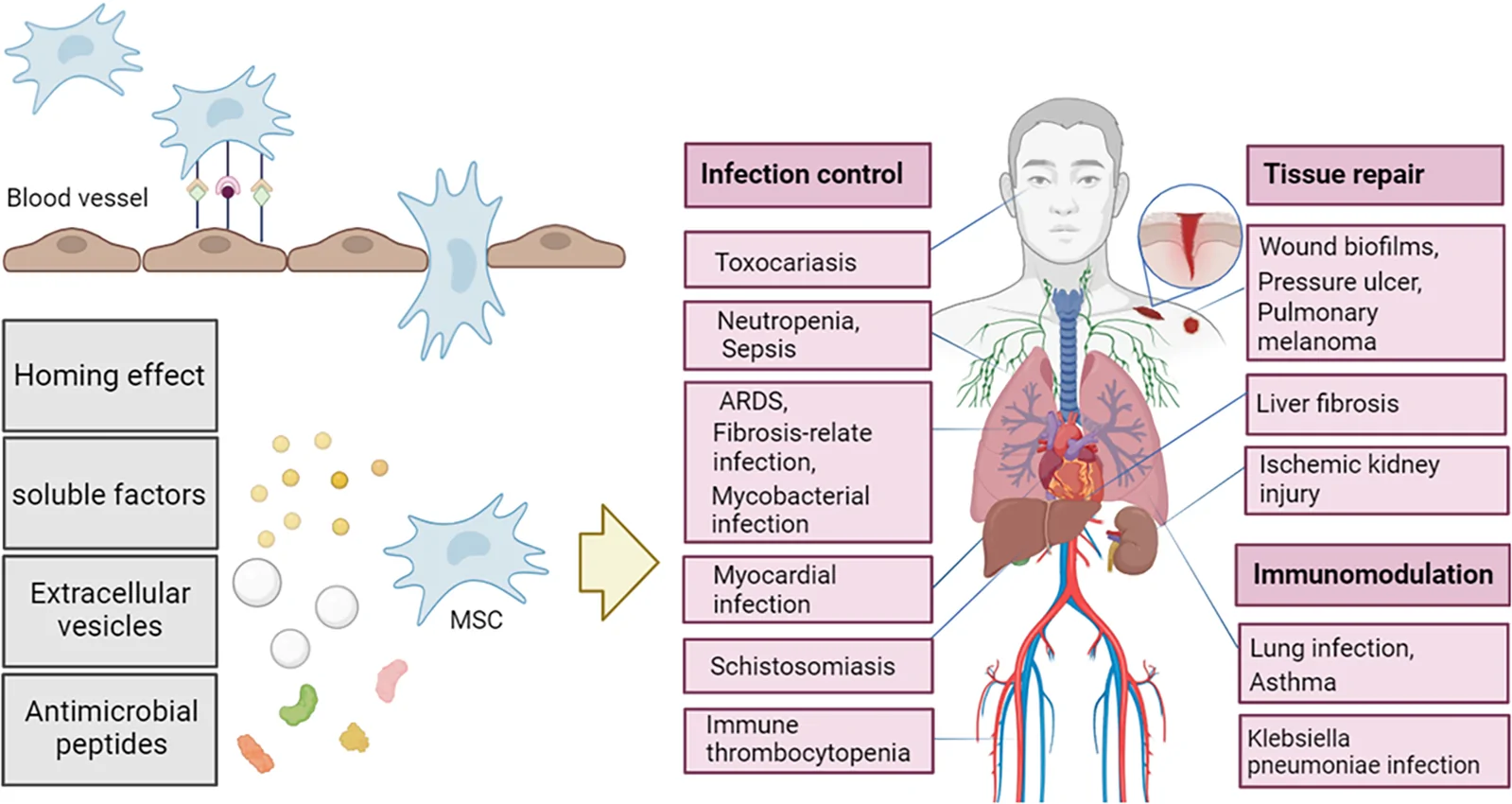
MSCs exhibit antimicrobial activity by homing effect in different infected, inflamed and damaged tissues. MSCs also provide therapeutic advantages by secreting soluble factors, extracellular vesicles, and antimicrobial peptides and proteins. MSC-based therapies exhibit various biological properties in combating a wide range of infectious diseases. Created in https://BioRender.com.

Unlike living cells, EVs preserve many of the therapeutic properties of MSCs while potentially mitigating safety concerns linked to cell-based therapies. Multiple studies have elucidated the critical role of EV-associated microRNAs (miRNAs) in mediating the antimicrobial and regenerative functions of MSCs. For instance, miR-223, miR-146a, and miR-199a modulate inflammatory pathways and facilitate tissue repair in conditions such as sepsis, hepatitis, and acute tissue injury (26). Similarly, EV-mediated delivery of miR-27a-3p fosters macrophage polarization toward an anti-inflammatory M2 phenotype and alleviates acute lung injury through the suppression of NF-κB signaling (27). Further evidence demonstrates that MSC-derived exosomes confer protection in viral myocarditis and periodontal disease via regulation of the AMPK/mTOR and p38 MAPK signaling pathways, respectively (28, 29). Additionally, MSC-derived exosomes influence autophagic pathways during infection; in viral myocarditis, umbilical cord MSC exosomes activate AMPK/mTOR-mediated autophagy flux, thereby reducing virus-associated myocardial injury. This underscores autophagy as a crucial mechanism through which EV cargo couples cellular stress responses to tissue protection (28).

Given that autophagy can either facilitate host defense or be subverted by pathogens depending on the infectious context, this pathway should be regarded as a context-dependent component of MSC-derived EV activity rather than a universally antiviral mechanism. Current evidence delineates three primary direct antimicrobial strategies employed by MSCs: (i) secretion of AMPs that directly target and neutralize pathogens; (ii) restriction of critical nutrients, such as iron, to limit pathogen proliferation; and (iii) delivery of antimicrobial and immunomodulatory cargo via EVs. These synergistic mechanisms underpin MSC-mediated antimicrobial activity and provide a compelling biological rationale for harnessing MSCs as innovative anti-infective agents, particularly in the face of rising antimicrobial resistance.

### 2.2 Immunomodulatory mechanisms

While direct antimicrobial activity plays a role in pathogen elimination, the most distinctive therapeutic property of MSCs resides in their capacity to modulate host immune responses. Importantly, MSCs are not inherently immunosuppressive; rather, their immunomodulatory phenotype is induced by specific environmental cues, notably inflammatory cytokines such as interferon-γ (IFN-γ), tumor necrosis factor-α (TNF-α), and interleukin-1β (IL-1β). This process, commonly referred to as “licensing,” prompts MSCs to secrete a diverse array of bioactive mediators, including prostaglandin E2 (PGE2), IDO, transforming growth factor-β (TGF-β), nitric oxide, and other soluble factors that collectively orchestrate innate and adaptive immune responses (30–33). Among these, IDO emerges as a pivotal dual-function mediator: inflammatory licensing induces tryptophan catabolism, which not only limits the proliferation of pathogens such as bacteria, viruses, fungi, and parasites but also suppresses hyperactive T-cell responses, thereby reshaping the local immune milieu (34, 35).

Through these mechanisms, MSCs mitigate excessive inflammation while preserving antimicrobial defense and facilitating tissue repair (**Figure 2**). A fundamental aspect of MSC-mediated immunomodulation involves the reprogramming of innate immune responses. During infection, MSCs orchestrate the recruitment, survival, and functional activation of neutrophils, monocytes, macrophages, and dendritic cells. Specifically, in bacterial infections, MSCs have been shown to enhance neutrophil survival and augment their bactericidal activity, thereby improving pathogen clearance (36). Concurrently, MSCs limit neutrophil-mediated tissue damage by decreasing the formation of neutrophil extracellular traps (NETs), which are increasingly recognized as key contributors to acute lung injury (ALI), sepsis, and cytokine storm syndromes. Recent evidence suggests that MSC-derived EVs can redirect NETosis toward apoptosis via reactive oxygen species/AKT-dependent pathways, thereby attenuating inflammation while maintaining host defense (37). Among innate immune cells, macrophages are prominent targets of MSC regulation. Multiple studies have demonstrated that MSCs promote the polarization of pro-inflammatory M1 macrophages toward the anti-inflammatory, tissue-reparative M2 phenotype, primarily through PGE2, TGF-β, and EV-mediated signaling pathways (38, 39). This immunomodulatory shift leads to decreased production of inflammatory cytokines, enhanced tissue remodeling, and improved clinical outcomes in conditions such as sepsis, ARDS, and chronic inflammatory infections. Importantly, macrophage reprogramming may serve as a common mechanism underlying the therapeutic effects of MSCs across a broad spectrum of infectious diseases, including bacterial, viral, fungal, and parasitic pathologies. In addition to their effects on macrophages, MSCs influence other innate immune regulators, notably antigen-presenting cells. Dendritic cells exposed to MSC-derived factors adopt a tolerogenic phenotype characterized by reduced maturation and heightened immunoregulatory capacity, mediated via pathways involving hepatocyte growth factor/Akt and Notch signaling (40, 41). Similarly, MSCs modulate natural killer (NK) cell responses in a context-dependent manner: during early viral infection, MSCs enhance NK cell cytotoxicity through the production of type I interferons, whereas sustained inflammation promotes the emergence of regulatory NK phenotypes driven by TGF-β and interleukin-6 (IL-6) signaling (42). These findings underscore the adaptive, environment-specific nature of MSC-mediated immune regulation. An additional emerging mechanism involves the modulation of the complement system, a pivotal component bridging innate and adaptive immunity. Excessive complement activation exacerbates tissue damage in severe infections, notably in viral illnesses such as COVID-19. MSC-derived exosomes have been demonstrated to inhibit the formation of the terminal complement complex (C5b-9), thereby attenuating NET formation, endothelial injury, and thromboinflammatory responses (43). Similarly, components of the MSC secretome play a crucial role in preserving endothelial glycocalyx integrity and mitigating complement-mediated vascular dysfunction during severe inflammatory conditions (44).

**Figure 2:**
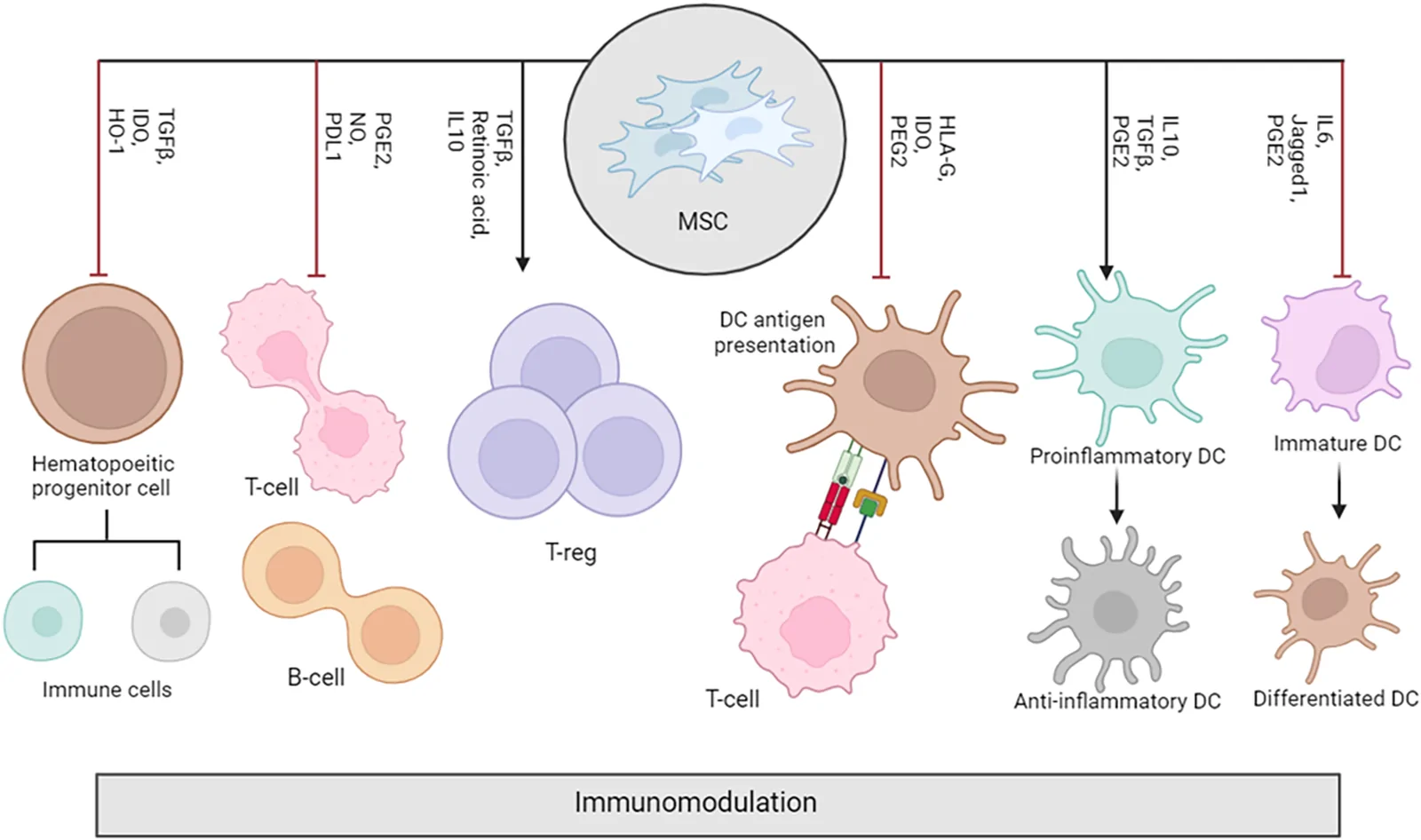
The immunomodulatory effects of MSCs have been demonstrated through various immune cell types. This includes Inhibition of progenitor cell differentiation into immune cells, the reduction of T-cell and B-cell proliferation and promotion of T-reg generation. Additionally, MSCs inhibit DC antigen presentation, induce an anti-inflammatory phenotype of DCs, and suppress the differentiation of immature DCs. Created in https://BioRender.com.

Beyond innate immunity, MSCs exert profound effects on adaptive immune responses. They regulate T-cell activation, differentiation, and cytokine production while promoting the expansion of regulatory T cells (Tregs). In experimental models of sepsis and acute lung injury, MSCs decrease Th1/Th2 and Th17/Treg ratios, thereby shifting the immune balance toward a controlled anti-inflammatory state and limiting tissue injury (45). Depending on the inflammatory context, MSCs can either suppress excessive T-cell responses or support protective immunity. For example, in simian immunodeficiency virus models, MSC administration enhanced antiviral immunity through improved germinal center formation, restoration of T follicular helper cells, enhanced antigen presentation, and increased virus-specific antibody production (46). Furthermore, MSCs can preserve B-cell survival and function through mitochondrial transfer and inhibition of inflammasome activation, illustrating an additional layer of immune regulation (47). Collectively, MSCs act as dynamic immune orchestrators that coordinate multiple components of innate and adaptive immunity rather than targeting a single immune pathway. Their ability to simultaneously enhance pathogen clearance, suppress excessive inflammation, and promote tissue repair likely explains their broad therapeutic potential across diverse infectious diseases. However, because these effects are highly dependent on MSC source, dose, timing of administration, and the prevailing inflammatory microenvironment, understanding the context-specific mechanisms governing MSC immunomodulation remains essential for the successful clinical translation of MSC-based anti-infective therapies.

### 2.3 Tissue repair as an antimicrobial strategy

Beyond their direct antimicrobial and immunomodulatory activities, MSCs promote host recovery through potent regenerative and tissue-protective mechanisms. Importantly, tissue repair should not be viewed merely as a secondary consequence of infection control. Increasing evidence suggests that restoration of tissue integrity represents an essential component of antimicrobial defense, as severe tissue injury itself can perpetuate inflammation, impair barrier functions, and facilitate pathogen persistence. By simultaneously limiting tissue damage and accelerating regeneration, MSCs help disrupt the self-sustaining cycle of infection, inflammation, and organ dysfunction. The regenerative effects of MSCs are mediated through multiple complementary mechanisms, including secretion of trophic factors, modulation of inflammatory responses, stimulation of angiogenesis, inhibition of fibrosis, and activation of endogenous repair pathways. These effects have been demonstrated across a wide range of infectious and inflammatory disease models affecting the lung, skin, liver, bone, and skeletal muscle.

In pulmonary infections and ALI, MSCs attenuate tissue damage through suppression of pro-inflammatory cytokines such as IL-1β, IL-6, and TNF-α while simultaneously promoting tissue remodeling and restoration of alveolar architecture. Experimental studies have demonstrated that MSCs improve lung function by reducing edema, hemorrhage, inflammatory infiltration, and fibrosis through modulation of pathways including TLR4/NF-κB/MAPK and TGF-β/Smad signaling (48). Furthermore, priming MSC-derived EVs with inflammatory cytokines such as IFN-γ and TNF-α further enhances their regenerative and immunomodulatory properties, providing promising results in both bacterial ALI and SARS-CoV-2-associated lung injury (49). The regenerative potential of MSCs is particularly evident in infected wound healing. In models of *Pseudomonas aeruginosa* burn wound infections, MSCs incorporated into collagen-fibrin scaffolds simultaneously reduced bacterial burden and accelerated tissue repair, demonstrating the close relationship between antimicrobial and regenerative functions (50). Similarly, MSC-derived secretome promoted wound closure and enhanced granulation tissue formation in methicillin-resistant *Staphylococcus aureus* infections while increasing the recruitment of neutrophils and reparative M2 macrophages (51). These findings suggest that successful pathogen clearance and tissue regeneration are tightly interconnected processes rather than independent therapeutic outcomes. MSCs also exhibit regenerative effects in chronic infectious diseases affecting internal organs. For example, disease-specific MSC secretome generated through exposure to hepatitis B virus-related inflammatory signals demonstrated anti-inflammatory, anti-apoptotic, and pro-regenerative effects in experimental liver injury. These effects were associated with reduced expression of inflammatory mediators and enhanced expression of regeneration-associated factors such as hepatocyte growth factor and proliferating cell nuclear antigen (52). Likewise, in parasitic diseases such as *Trichinella spiralis*-induced myopathy, MSC administration improved muscle regeneration, restored motor function, and reduced inflammatory infiltration and fibrosis (53). Recent studies further suggest that enhancing the antimicrobial properties of MSCs can simultaneously amplify their regenerative potential. Preconditioning MSCs with the antimicrobial peptide nisin increased expression of LL-37 and hepcidin, resulting in improved bacterial clearance, reduced inflammation, and accelerated wound repair in multidrug-resistant *P. aeruginosa* infections (54). Similarly, MSC-derived exosomes promoted bone regeneration and reduced inflammation in *S. aureus*-induced osteomyelitis through activation of osteogenic pathways and suppression of inflammatory cytokines (55). Collectively, these findings indicate that tissue repair represents a fundamental component of MSC-mediated antimicrobial defense. By preserving tissue architecture, restoring barrier functions, and preventing chronic inflammatory damage, MSCs create a microenvironment that favors pathogen clearance and functional recovery. This dual capacity to control infection while promoting regeneration distinguishes MSC-based therapies from conventional antimicrobial treatments and may explain their therapeutic potential across diverse infectious diseases.

## 3 Preclinical and clinical applications of MSCS in infectious diseases

Accumulating preclinical and clinical evidence indicates that MSCs hold significant therapeutic potential across a broad spectrum of infectious diseases. However, the magnitude and reproducibility of their effects vary markedly depending on the pathogen involved. While bacterial infections typically demonstrate robust and consistent responses, viral, fungal, and parasitic infections tend to yield more heterogeneous outcomes. These disparities suggest that MSC efficacy is heavily modulated by pathogen-specific biology, the immune microenvironment, and the dominant pathogenic mechanisms driving disease progression. A comprehensive understanding of these pathogen-specific responses is crucial for identifying optimal clinical applications and for the rational development of MSC-based therapeutic strategies with enhanced efficacy and safety.

### 3.1 Why do MSCs perform best in bacterial infections?

Among all infectious diseases investigated to date, bacterial infections provide the strongest evidence supporting MSC-based therapies. Across multiple preclinical models and early clinical studies, MSCs consistently improve bacterial clearance, reduce excessive inflammation, and promote tissue repair. These beneficial effects appear to result from the convergence of three complementary mechanisms: direct antimicrobial activity through antimicrobial peptides, enhancement of innate immune responses, and acceleration of tissue regeneration. The most compelling evidence comes from pulmonary infections and sepsis, where MSC administration improves outcomes in experimental pneumonia caused by *P. aeruginosa*, *Klebsiella pneumoniae*, and *Escherichia coli* (56–58). In these models, MSCs enhance bacterial phagocytosis, support neutrophil and macrophage antimicrobial functions, and limit inflammatory tissue injury. Importantly, these preclinical findings have been partially translated into the clinical setting, where MSCs have demonstrated favorable safety profiles and encouraging signals of efficacy in severe community-acquired pneumonia and sepsis (59–61). Similarly, encouraging results have been reported in chronic bacterial infections, including tuberculosis and nontuberculous mycobacterial disease. MSC-derived EVs suppress bacterial growth and inflammatory responses in *Mycobacterium tuberculosis* infection, while bone marrow-derived MSCs have demonstrated antimicrobial and antibiotic-enhancing effects against *Mycobacterium avium* and *Mycobacterium intracellulare* (62, 63). These observations suggest that MSCs may represent valuable adjunctive therapies in infections where inflammation contributes substantially to pathology.

The therapeutic benefit of MSCs is particularly evident in wound-associated infections. Numerous studies have demonstrated accelerated wound closure, reduced bacterial burden, enhanced angiogenesis, and improved tissue remodeling in infections caused by *S. aureus*, *P. aeruginosa*, and other multidrug-resistant pathogens. Interestingly, biomaterial-assisted delivery systems and MSC-derived secretomes often further enhance these effects, highlighting the growing interest in cell-free therapeutic approaches (50, 64–67). Nevertheless, bacterial infections also reveal important limitations. Implant-associated infections illustrate that MSC-pathogen interactions are not universally beneficial. For example, MSCs may facilitate pathogenic behavior in *Cutibacterium acnes*, potentially contributing to chronic osteomyelitis and biofilm-associated infections (68, 69). These findings emphasize that bacterial species, biofilm formation, and local microenvironmental factors critically influence therapeutic outcomes. Taken together, current evidence suggests that bacterial infections represent the most advanced and promising indication for MSC-based antimicrobial therapies. The combination of direct antimicrobial activity, immune modulation, and tissue repair appears particularly effective against extracellular bacterial pathogens, making bacterial sepsis, pneumonia, and chronic wound infections leading candidates for future clinical translation.

### 3.2 Why are MSC responses more variable in viral infections?

Unlike bacterial infections, where pathogen clearance and tissue repair frequently occur in parallel, viral diseases present a considerably more complex therapeutic landscape. Across numerous preclinical and clinical studies, MSCs consistently reduce inflammation, attenuate tissue damage, and promote tissue regeneration. However, their effects on viral replication, survival, and long-term clinical outcomes remain far more heterogeneous. This distinction suggests that MSCs primarily target virus-induced immunopathology rather than directly eliminating viral pathogens. Consequently, their therapeutic efficacy depends largely on the relative contribution of immune-mediated tissue injury versus uncontrolled viral replication during disease progression.

#### 3.2.1 Mechanisms underlying variable antiviral efficacy

The antiviral efficacy of MSCs is inherently variable and results from a complex interplay of multiple determinants. Viral strain and tropism critically influence whether MSCs or their derivatives encounter susceptible target tissues and whether the pathogen can directly modulate MSC function. Timing of administration is equally crucial; treatment during phases characterized by hyperinflammation may enhance immunomodulatory benefits, whereas intervention during periods of uncontrolled viral replication may yield limited antiviral effects if MSC-mediated direct antiviral activity is insufficient. Additional factors, including disease severity, viral inoculum, MSC tissue origin, dosage, route of administration, and inflammatory licensing, further influence the delicate balance between antiviral immunity and tissue-protective immunosuppression. Collectively, the most effective therapeutic window for MSC intervention is likely when immune-mediated tissue injury predominates as a driver of morbidity, and MSC therapy can attenuate pathogenic inflammation without impairing essential antiviral host defenses.

#### 3.2.2 COVID-19: the largest clinical evidence base with variable efficacy on hard outcomes

The preponderance of clinical evidence supporting MSC therapy has primarily arisen from investigations during the COVID-19 pandemic. Mechanistic studies and early clinical trials indicate that MSCs and MSC-derived EVs can attenuate inflammatory signaling, maintain immune homeostasis, and facilitate pulmonary repair in severe SARS-CoV-2 infections. These therapeutic effects are mediated via multiple complementary mechanisms, including modulation of cytokine profiles, delivery of antiviral microRNAs, and promotion of tissue regeneration. Experimental data demonstrate that MSC-derived EVs express several antiviral microRNAs capable of regulating inflammatory pathways and disrupting SARS-CoV-2 infection. Additionally, priming MSCs with poly(I:C) enhances their antiviral and immunomodulatory capacities by inhibiting viral entry and augmenting immune regulation (70–72). Further studies suggest that lyophilized MSC-derived EVs may impede viral entry and mitigate COVID-19-associated inflammation through JAK/STAT pathway modulation (73, 74). While these mechanistic insights and biomarker data are compelling, they are more consistent than evidence from definitive clinical outcomes. Phase I and II trials generally demonstrate feasibility and a favorable short-term safety profile, with some reports indicating improvements in inflammatory biomarkers, oxygenation levels, and specific recovery metrics (75–77). However, randomized studies have not consistently demonstrated benefits on primary clinical endpoints. Gorman et al. (78) reported no improvements in lung function, survival, or long-term outcomes; Lightner et al. (79) found no significant primary mortality benefit associated with MSC-derived EVs, despite signals in exploratory subgroup analyses; and Yuan et al. (80) observed no significant improvements in lung imaging, pulmonary function, or long-COVID symptoms, although overall health status improved at long-term follow-up. Consequently, COVID-19 currently provides the most substantial clinical evidence base for MSC-based therapies, yet it does not offer unequivocal proof of efficacy concerning mortality or other definitive outcomes. Ongoing clinical trials are assessing MSC therapy for long COVID and viral pneumonia (NCT06156241, NCT06492798, NCT06416709, NCT06693362), reflecting increasing translational interest in MSC-based antiviral interventions.

#### 3.2.3 Influenza highlights the context-dependent nature of MSC therapy

Influenza virus infection illustrates the variability of MSC therapeutic responses. Across H1N1, H5N1, H7N9, and H9N2 models, MSC administration consistently reduces pulmonary inflammation, cytokine production, and acute lung injury. However, effects on viral replication and survival differ substantially between studies. For example, MSCs attenuated lung injury in H1N1 and H5N1 infections without significantly improving mortality, whereas more pronounced survival benefits were observed in H7N9 and H9N2 models (81–83). Similarly, Wharton’s jelly-derived MSC-derived EVs reduced inflammatory responses during co-infection with SARS-CoV-2 and H1N1 through the miR-146a/NF-κB pathway, while IL-18-primed MSCs enhanced protection against severe viral pneumonia (84–86). These apparently contradictory findings likely reflect differences in viral strain, inoculum, timing of MSC administration, disease severity, and the relative importance of immune-mediated pathology. Collectively, they suggest that MSCs are most effective in viral infections where excessive host inflammation, rather than viral replication alone, drives disease severity.

#### 3.2.4 Beyond respiratory viruses: expanding therapeutic applications

The therapeutic potential of MSCs extends beyond respiratory viruses. In cytomegalovirus infection, MSC-derived exosomes reduce pulmonary inflammation and fibrosis through inhibition of NF-κB/NLRP3 signaling, while MSCs have also demonstrated protective effects against murine gamma-herpesvirus pneumonia by reducing viral burden and tissue injury (87, 88). In Zika virus infection, however, a different interaction emerges. Human umbilical cord-derived MSCs are themselves susceptible to Zika virus infection, which alters mitochondrial function and suppresses antiviral miR-142-5p expression to facilitate viral replication. Conversely, IFN-β-primed MSC-derived EVs overexpressing miR-431-5p effectively suppress viral replication, illustrating how engineering or priming strategies may overcome virus-induced MSC dysfunction (89, 90). Similarly, genetically engineered MSCs have been explored as both therapeutic and vaccine platforms. Engineered MSCs expressing ACE2 act as decoy cells capable of neutralizing SARS-CoV-2 (91), whereas MSCs expressing hepatitis C virus non-structural proteins have demonstrated promising vaccine potential by enhancing virus-specific cellular immunity in experimental models (92–94). These studies illustrate the expanding role of MSCs beyond conventional cell therapy toward multifunctional antiviral delivery platforms.

#### 3.2.5 Clinical translation remains heterogeneous

Clinical outcomes beyond COVID-19 remain inconsistent. In chronic HIV infection, randomized clinical trials have yielded conflicting results: one study reported no significant immune recovery and noted thrombosis in three of five MSC-treated patients, a notable safety signal in this small cohort, while another suggested improved immune reconstitution with a lower-dose umbilical cord-derived MSC regimen (95, 96). Similar investigations are underway for hepatitis B cirrhosis, HPV infection, Long COVID, and AIDS, underscoring ongoing clinical interest despite the variability observed across different viral diseases (NCT03826433, NCT06610773, NCT06156241, NCT05872659, NCT05939167).

#### 3.2.6 Critical perspectives

Collectively, current evidence indicates that MSCs exert their greatest therapeutic benefit in viral diseases primarily by controlling excessive inflammation and promoting tissue regeneration, rather than by directly suppressing viral replication. This distinction accounts for the more consistent mechanistic and biomarker signals observed in inflammatory syndromes such as COVID-19-associated ARDS, despite mixed effects on hard clinical endpoints and variability across influenza, HIV, hepatitis, and other viral infections. Future research should therefore focus on identifying viral diseases in which immunopathology is the predominant driver of disease severity, as these conditions are likely to benefit most from MSC-based therapies. Concurrently, advances in MSC priming, genetic engineering, and extracellular vesicle technologies hold promise for further enhancing antiviral efficacy and developing pathogen-specific MSC therapeutics.

### 3.3 The fungal paradox: when MSCs protect or promote fungal persistence

Fungal infections present one of the most complex contexts for MSC-based therapies. Unlike bacterial infections, where MSCs generally exert beneficial antimicrobial effects, interactions between MSCs and fungal pathogens are highly context dependent and may result in either protection or enhanced fungal persistence. This duality, herein referred to as the “fungal paradox,” highlights the need to understand pathogen-specific MSC responses before clinical translation. On one hand, MSCs demonstrate significant antifungal and tissue-protective properties. Studies involving *Candida albicans*, *Aspergillus fumigatus*, and *Fusarium* species have shown that MSCs can inhibit fungal growth, reduce inflammation, and promote tissue repair through the secretion of antimicrobial peptides such as LL-37, immunomodulatory factors, and EVs. In models of candidiasis, MSC-derived LL-37 directly suppresses fungal growth, while in aspergillosis and fungal keratitis, MSC administration attenuates inflammation and preserves tissue integrity (97–100). Similarly, MSC-derived EVs mitigate oxidative stress and tissue injury induced by fungal toxins, further supporting their therapeutic potential (101, 102). However, not all fungi are susceptible to MSC-mediated control. Studies involving *Paracoccidioides brasiliensis* and *Histoplasma capsulatum* reveal a fundamentally different interaction. Rather than being eliminated, these intracellular fungi can survive within MSCs, alter MSC differentiation and immunological functions, and potentially exploit MSCs as protective reservoirs. Such findings suggest that some fungal pathogens may hijack the immunomodulatory properties of MSCs to evade immune surveillance and promote chronic infection (103, 104). These contrasting outcomes indicate that fungal susceptibility to MSC-mediated therapy depends largely on pathogen biology. Extracellular fungi appear more likely to be controlled through MSC-derived antimicrobial and immunoregulatory mechanisms, whereas intracellular or immune-evasive fungi may exploit MSCs as survival niches. Consequently, fungal infections should not be considered a single therapeutic category, and future studies must identify the specific fungal characteristics that predict beneficial versus detrimental MSC responses. Taken together, current evidence supports the therapeutic potential of MSCs in selected fungal diseases while simultaneously highlighting important safety concerns. Defining the mechanisms that govern the balance between fungal clearance and fungal persistence remains a critical prerequisite for clinical translation.

### 3.4 The parasite dilemma: host protection versus parasite adaptation

Parasitic diseases remain major causes of morbidity and mortality worldwide, particularly in tropical and subtropical regions, where chronic infection often leads to persistent inflammation, fibrosis, immune dysregulation, and irreversible organ damage. Although conventional antiparasitic drugs effectively reduce parasite burden in many cases, they frequently fail to reverse tissue injury or restore immune homeostasis. Consequently, MSCs have attracted increasing interest as adjunctive therapies because of their potent immunomodulatory, anti-inflammatory, anti-fibrotic, and regenerative properties. However, unlike bacterial infections, where MSC-mediated antimicrobial activity is relatively consistent, parasite–MSC interactions are considerably more complex. Accumulating evidence indicates that MSCs primarily improve host pathology rather than directly eliminating parasites, while several parasites can actively modify MSC phenotype and function. This duality, referred to here as the “parasite dilemma,” highlights both the therapeutic promise and the biological complexity of MSC-based therapies in parasitic diseases. Different mechanisms and effects of MSCs against various pathogens are summarized in **Table 1**.

**Table 1:** Overview of MSCs antimicrobial mechanisms, effects, and clinical translation status.

Pathogen class	Primary mechanism of MSC benefit	Effect on pathogen clearance	Clinical maturity
Bacteria	Direct antimicrobial + innate immunity	Strong	High
Viruses	Immunomodulation + tissue repair	Variable	Moderate
Fungi	Context-dependent	Variable	Low
Parasites	Immunoregulation + regeneration	Limited/variable	Low

The “Effect on pathogen clearance” classification primarily summarizes preclinical mechanistic and efficacy evidence. Clinical maturity is shown separately and should not be interpreted as evidence of proven clinical efficacy.

#### 3.4.1 Tissue protection is the predominant therapeutic mechanism

A recurring observation across experimental models is that MSCs primarily reduce inflammation, fibrosis, and tissue injury while promoting regeneration. In malaria, MSC therapy enhances erythropoiesis and lymphopoiesis, contributing to improved host recovery (105). Similarly, in experimental cerebral malaria, MSC administration does not significantly alter parasite burden but protects against blood–brain barrier disruption, vascular inflammation, and neurological dysfunction (106). These findings illustrate that MSCs act predominantly by limiting immune-mediated pathology rather than directly targeting the parasite. The same principle is observed in Chagas disease, where genetically engineered MSCs overexpressing insulin-like growth factor-1 (IGF-1) or granulocyte colony-stimulating factor (G-CSF) significantly reduce cardiac fibrosis and improve tissue repair while maintaining their immunomodulatory properties (107, 108). Likewise, in schistosomiasis, MSCs and MSC-derived EVs consistently attenuate hepatic fibrosis, suppress inflammatory responses, and improve liver function, particularly when combined with praziquantel therapy (109–112). Collectively, these studies indicate that regeneration and fibrosis control constitute the dominant therapeutic mechanisms of MSCs in chronic parasitic diseases.

#### 3.4.2 Leishmaniasis illustrates the potential of adjunct MSC therapy

Among parasitic diseases, cutaneous leishmaniasis provides one of the strongest preclinical rationales for MSC therapy. Multiple independent studies have demonstrated reduced lesion size, decreased parasite burden, enhanced nitric oxide production, increased IFN-γ secretion, and a shift toward protective Th1 immune responses following administration of adipose- or bone marrow-derived MSCs (113–116). Furthermore, MSC-derived secretome and EVs also exhibit anti-leishmanial activity while accelerating wound healing (117, 118). Interestingly, combining MSCs with conventional antileishmanial drugs has produced mixed outcomes. While synergistic therapeutic effects have been reported with meglumine antimoniate in some experimental models (119), other studies failed to demonstrate additional benefit over MSC therapy alone (120). These discrepancies suggest that the efficacy of combination therapies depends on the mechanisms of action of both the antiparasitic agent and the MSC product, highlighting the need for pathogen-specific optimization.

#### 3.4.3 Parasites actively reshape the biology of MSCS

Unlike bacterial pathogens, several parasites directly influence MSC phenotype and function. *Leishmania major*, for example, reprograms MSCs from an immunosuppressive toward a pro-inflammatory phenotype, potentially altering their therapeutic properties (121). Likewise, antigens derived from *Echinococcus granulosus* impair MSC migration, modify cytokine secretion, and alter extracellular vesicle microRNA composition, thereby affecting immune regulation and osteogenic differentiation (122, 123). These observations demonstrate that parasites are not passive targets of MSC therapy but can actively manipulate MSC biology to favor their own persistence. A similar concern has emerged in malaria. Direct co-culture experiments revealed that MSCs and their conditioned medium increase the infectivity of *Plasmodium falciparum* gametocytes in mosquitoes, suggesting that MSC-derived factors may inadvertently enhance parasite transmission under certain conditions (124). Such findings underscore the importance of understanding host–parasite–MSC interactions before clinical application.

#### 3.4.4 Clinical translation remains limited

Despite encouraging experimental evidence, clinical translation remains at an early stage. The first reported clinical application involved a pediatric patient with toxoplasmic encephalitis following allogeneic hematopoietic stem cell transplantation, in whom combined antiparasitic therapy and umbilical cord-derived MSCs resulted in parasite clearance and complete neurological recovery (125). However, beyond isolated case reports, clinical evidence remains scarce. Most studies are still confined to preclinical models, and robust clinical trials evaluating safety, efficacy, optimal dosing, and long-term outcomes are lacking.

An overview of the most relevant clinical trials is presented in **Table 2**.

**Table 2:** Summary of MSC- based clinical trials across different infectious diseases.

Trial registry ID	Phase	Disease/condition	Pathogen	MSC source	Dose and route of administration	Main outcomes	Enrolment	Status	References
NCT03158727	I/II	Severe community-acquired bacterial pneumonia	Bacterial pneumonia	Allogeneic AT-MSCs	1.6 × 10^8^ cells × 2 IV infusions (day 1 & 3)	No significant safety difference in AEs vs. placebo. No significant efficacy in cure rate, ventiltor-free days and survival but showed modulated immune, coagulation and apoptosis biomarkers	83	Completed	(60, 61)
IRCT20200217046526N2	II	ARDS	SARS-CoV-2	Allogeneic MSCs derived from perinatal tissue	1×10^8^ cells × 2 consecutive IV infusions 1×10^8^ cells × 1 IV infusion and 1 dose of EVs via nebulization (isolated from 2×10^8^ cells)	No serious AEs. Significant reduction in inflammatory markers	43	Completed	(76)
NCT05019287	I/II	Severe COVID-19	SARS-CoV-2	Allogeneic menstrual blood stromal cells	5 ml of secretome × 5 IV infusions (5 consecutive days)	No treatment-related AEs. Improved survival, oxygenation, pulmonary involvement and immune recovery and downregulated cytokine storm	30	Completed	(75)
NCT03042143	I/II	Moderate to severe ARDS	SARS-CoV-2	Allogeneic UC-MSCs	4× 10^8^ cells × 1 IV infusion	No significant safety difference in AEs vs. placebo. No improvement in lung function, survival or long-term outcomes	59	Active, not recruiting	(78)
NCT04493242	II	Moderate to severe ARDS	SARS-CoV-2	BM-MSCs	10 ml of EVs × 2 IV infusions (day 1 & 4) 15ml of EVs × 2 IV infusions (day 1 & 4)	No treatment-related AEs. 15ml dose showed no significant primary mortality benefit but reduced 60-day mortality risk in exploratory subgroups and improved ventilation-free days	102	Completed	(79)
IRCT20210919052520N1	0	ARDS	SARS-CoV-2	Allogeneic AT-MSCs	2 ml of secretome × 2 doses via nebulization (5 consecutive days)	No treatment-related AEs. Faster ICU recovery, improved oxygenation and reduced inflammation	20	Completed	(77)
NCT06156241	I	Post-acute Covid-19 syndrome	SARS-CoV-2	Allogeneic CB-MSCs	4×10^6^/6×10^6^/8×10^6^ and 10×10^6^ cells/kg (dose-escalation) × 1 IV infusion	N/A	12	Unknown	
NCT06492798	I/II	Post-acute Covid-19 syndrome	SARS-CoV-2	UC- MSCs	1×10^6^ cells/kg × 1 IV infusion (with possible 2^nd^ dose) 2 doses of exosomes via nebulization (5 days)	N/A	76	Recruiting	
NCT06416709	I/II	Lung injury	Viral pneumonia	MSCs	5×10^7^ cells/10 mL × 3 IV infusions in total once every 3 days	N/A	80	Unknown	
NCT06693362	I	Severe viral pneumonia	Viral pneumonia (COVID-19 excluded)	Allogeneic MB-MSCs	3×10^7^ cells × 3 IV infusions (day 1, 3 & 5)	N/A	60	Recruiting	
ChiCTR2000029990	I/II	COVID-19 pneumonia	SARS-CoV-2	Allogeneic MSCs	1×10^6^ cells/kg × 1 IV infusion	No treatment-related AEs. Improved clinical symptoms and reduced inflammatory markers, regulated immune responses and enhanced viral clearance	7		(126)
NCT04288102	II	Severe COVID-19	SARS-CoV-2	Allogeneic UC-MSCs	- 4×10^6^ cells/kg × 3 IV infusions (day 0, 3 & 6)	No treatment-related AEs. No significant benefit in lung imaging, function and long COVID symptoms but with significant improvement in general health at 3-year follow-up	100	Completed	(80)
NCT02290041	I/II	HIV-infected immune Nonresponders	HIV-1	Allogeneic AT-MSCs	- 1×10^6^ cells/kg × 4 IV infusions (week 0, 4, 8 & 20)	A raised safety concern (thrombosis in 3/5 patients) and no improvement in immune recovery or reduced inflammation	15	Completed	(95)
NCT01213186	II	Chronic HIV-infected immune nonresponders	HIV-1	Allogeneic UC- MSCs	0.5×10^6^ cells/kg × 6 IV infusions (week 0, 4, 12, 24, 36 & 48) 1.5×10^6^ cells/kg × 6 IV infusions (week 0, 4, 12, 24, 36 & 48)	No significant safety difference in AEs vs. placebo. No significant improvement in immune recovery with the low dose performed somewhat more effective	72	Unknown	(96)
NCT05872659	I	HIV-infected immune nonresponders	HIV	UC-MSCs	1×10^6^ cells/kg × 3 IV infusions (day 0, 30 & 60)	N/A	20	Unknown	
NCT05939167	I/II	Late stage HIV-infected patients	HIV	UC-MSCs	0.75–1.0×10^6^ cells/kg × 3 IV infusions (week 0, 2 & 4) 0.75–1.0×10^6^ cells/kg × 6 IV infusions (week 0, 2, 4, 24, 26, 28)	N/A	150	Recruiting	
NCT03826433	I	Hepatitis B cirrhosis	HBV	UC-MSCs	6×10^7^ cells × IV infusion	N/A	20	Recruiting	
NCT06610773	I/II	High-risk HPV infection	HPV	UC-MSCs	1×10^6^ cells/kg × IV infusion	N/A	60	Recruiting	

Clinical-trial inclusion criteria and baseline severity varied across studies. In general, enrolled populations met the disease- and protocol-specific definitions shown in the Disease/Condition column (e.g., moderate-to-severe or severe COVID-19/ARDS, severe community-acquired bacterial pneumonia, or HIV-infected immune non-responders). Because detailed exclusion criteria were not consistently reported in the sources summarized here, readers should consult the original trial reports or registry records for complete eligibility criteria and baseline severity definitions.

AT-MSCs, Adipose tissue derived mesenchymal stem cells, CB-MSCs, Cord blood derived mesenchymal stem cells IV; Intravenous; AEs, Adverse events; ARDS, Acute respiratory distress syndrome; EVs, Extracellular vesicles, MB-MSCs, Menstrual blood stromal cells, UC-MSCs, Umbilical cord-derived MSCs, BM-MSCs, Bone marrow-derived MSCs; HPV, Human papillomavirus; HIV, Human Immunodeficiency Virus; HBV, Hepatitis B virus.

#### 3.4.5 Critical perspectives

Collectively, parasitic diseases reveal that MSCs should not be considered conventional antiparasitic agents. Their principal therapeutic value lies in restoring immune homeostasis, reducing fibrosis, and promoting tissue regeneration, while direct effects on parasite elimination remain variable. Moreover, the ability of several parasites to reprogram MSC phenotype or exploit MSC-derived factors illustrates the bidirectional nature of host–parasite–MSC interactions. Future research should therefore focus on identifying the molecular mechanisms governing these interactions and determining which parasitic diseases are most likely to benefit from pathogen-tailored MSC therapies. Such knowledge will be essential for safely translating MSC-based interventions into clinical parasitology.

## 4 Determinants of antimicrobial peptide production in MSCS

The antimicrobial activity of MSCs is not constitutive but is dynamically regulated by the surrounding microenvironment. Exposure to microbial products, inflammatory cytokines, hypoxia, and tissue-specific signals collectively shapes the production of AMPs, thereby determining the antimicrobial potency of MSCs. However, current evidence also indicates that AMP regulation is highly context-dependent and varies according to MSC source, culture conditions, and the nature of the infectious stimulus.

### 4.1 Microbial and inflammatory licensing of AMP production

Recognition of pathogens represents one of the principal mechanisms driving AMP production in MSCs. Pattern-recognition receptors, particularly Toll-like receptors and nucleotide-binding oligomerization domain -like receptors, detect pathogen-associated molecular patterns and activate signaling pathways that stimulate AMP secretion (127). Besides direct secretion, AMPs may also be delivered through EVs or released as part of the MSC secretome, thereby extending their antimicrobial activity beyond the producing cells (8). Among MSC-derived antimicrobial peptides, LL-37 remains the best-characterized example. Multiple studies have demonstrated that microbial exposure significantly enhances LL-37 expression (128). One of its major regulatory pathways involves vitamin D signaling, as the LL-37 promoter contains multiple vitamin D response elements. Accordingly, treatment of adipose-derived MSCs with 1,25-dihydroxyvitamin D3 rapidly increases LL-37 expression and antimicrobial activity, whereas inhibition of the vitamin D receptor abolishes this response (129). LL-37 production can also be enhanced through TLR3 activation using poly(I:C), particularly under low-serum culture conditions, highlighting the influence of culture media composition on AMP detection and secretion (130). Inflammatory cytokines similarly regulate AMP production. HAMP, a key mediator of nutritional immunity, is induced in bone marrow-derived MSCs following exposure to TNF-α, IFN-γ, IL-6, or Gram-negative bacteria (131). Interestingly, the regulatory mechanisms differ from those described in hepatocytes, where IL-6 alone predominates, suggesting that stromal cells employ distinct signaling pathways to integrate inflammatory cues. Likewise, bacterial stimulation increases hepcidin expression in murine sepsis models following exposure to *Staphylococcal enterotoxin B*, with LL-37 exhibiting a similar expression profile (132).

Other antimicrobial peptides exhibit their own regulatory pathways. β-defensin-2 is primarily induced through TLR4 signaling following bacterial challenge (18), whereas LCN2 expression increases under inflammatory conditions and contributes to iron sequestration during infection (8). Collectively, these findings indicate that AMP induction is coordinated through multiple pathogen-recognition and inflammatory pathways rather than a single universal regulatory mechanism.

### 4.2 Tissue origin and culture conditions influence AMP production

An emerging concept is that AMP production varies substantially according to MSC tissue origin. Menstrual blood-derived MSCs, for example, do not significantly increase LL-37 expression following bacterial stimulation, whereas bone marrow-derived MSCs display robust induction of both LL-37 and hepcidin under similar conditions (132, 133). Likewise, equine endometrial MSCs express markedly higher levels of LCN2 than bone marrow- or adipose-derived MSCs, probably reflecting the continuous microbial exposure experienced by endometrial tissues (134). These observations suggest that tissue-specific adaptation contributes significantly to the antimicrobial phenotype of MSCs. Culture conditions further influence AMP production. Serum concentration represents one important variable because fetal bovine serum can bind cationic peptides such as LL-37, thereby reducing their measurable extracellular concentration (130). Such methodological differences may partly explain the variability reported among studies and emphasize the importance of standardized culture conditions for evaluating MSC antimicrobial potency.

### 4.3 AMP regulation remains incompletely understood

Although microbial and inflammatory stimuli generally enhance AMP expression, not all studies report improved antimicrobial activity. For example, stimulation of MSCs with CpG oligonucleotides, a TLR9 agonist, markedly increased transcription of LL-37, β-defensin-2, hepcidin, surfactant protein D, and lipocalin genes without significantly enhancing bactericidal activity (135). These findings suggest that increased gene expression does not necessarily translate into greater antimicrobial function and raise the possibility that post-transcriptional regulation, peptide processing, or secretion efficiency may represent additional limiting factors. Moreover, MSCs regulate antimicrobial defenses not only through direct AMP secretion but also indirectly by stimulating AMP production in neighboring cells. For example, MSC-derived CCL2 chemokine induces keratinocytes to upregulate cathelicidin and β-defensin expression, illustrating how MSCs amplify local antimicrobial responses through paracrine communication (136).

### 4.4 Future perspectives

Despite substantial progress in identifying MSC-derived AMPs, the molecular mechanisms governing their regulation remain poorly understood. In particular, little is known about the genetic, epigenetic, and metabolic networks controlling AMP expression, secretion, and functional activity. Understanding these regulatory mechanisms represents an important research priority because they may provide opportunities to enhance the antimicrobial potency of MSCs through pharmacological priming, genetic engineering, or synthetic biology approaches. Future studies should also aim to establish standardized potency assays linking AMP expression profiles with antimicrobial efficacy against specific pathogens. Such efforts could ultimately support the development of a comprehensive MSC antimicrobial peptide atlas, integrating peptide expression, regulatory pathways, microbial targets, and therapeutic performance. This resource would facilitate rational design of pathogen-specific MSC products and accelerate the clinical translation of next-generation cell-based and cell-free anti-infective therapies.

## 5 From experimental promise to clinical translation: remaining challenges

Despite the encouraging preclinical and early clinical evidence supporting MSC-based therapies for infectious diseases, their widespread clinical implementation remains limited. The discrepancy between promising experimental results and relatively slow clinical translation reflects a series of interconnected biological, manufacturing, and regulatory challenges. Rather than representing independent obstacles, these factors collectively determine the reproducibility, safety, and therapeutic efficacy of MSC-based products.

### 5.1 Biological heterogeneity remains the principal challenge

One of the greatest sources of variability in MSC therapy is the intrinsic heterogeneity of the cells themselves. MSC phenotype and function are strongly influenced by donor characteristics, tissue source, isolation procedures, expansion protocols, and prolonged *in vitro* culture (137–140). Consequently, MSC products generated by different laboratories often exhibit substantial differences in proliferation capacity, immunomodulatory activity, antimicrobial potency, and regenerative potential. This biological variability contributes significantly to the inconsistent therapeutic outcomes reported across preclinical and clinical studies and highlights the urgent need for standardized manufacturing procedures and potency assays. In addition to heterogeneity, prolonged ex vivo expansion may induce cellular senescence, chromosomal instability, altered differentiation potential, and reduced therapeutic efficacy. Although malignant transformation of clinical-grade MSCs appears uncommon, genomic stability should be routinely evaluated before clinical application to minimize potential safety risks (138).

### 5.2 Manufacturing and quality control require further standardization

Manufacturing conditions profoundly influence MSC quality and therapeutic performance. Cell contamination remains an important concern, particularly during prolonged culture, where viral, bacterial, or mycoplasma contamination may occur either through donor tissues or during cell processing (138, 141). Detection of latent viral DNA in human bone marrow further emphasizes the importance of rigorous donor screening and Good Manufacturing Practice -compliant production workflows (142). These considerations are particularly relevant for anti-infective applications, where contamination could compromise both product safety and therapeutic efficacy. Culture supplements also represent an unresolved challenge. Fetal bovine serum, although widely used for MSC expansion, carries risks of xenogeneic pathogen transmission, batch-to-batch variability, and unwanted immunogenicity (143–145). Moreover, fetal bovine serm may interfere with the detection and activity of antimicrobial peptides such as LL-37 by binding cationic peptides during culture (130). Human platelet lysate has emerged as a promising alternative and is currently recommended by the International Society for Cell & Gene Therapy because of its ability to support MSC proliferation while reducing xenogeneic risks (146, 147). Nevertheless, whether serum-free or humanized culture systems fully preserve the antimicrobial properties of MSCs remains uncertain and deserves further investigation.

### 5.3 Optimizing the dose, timing, and route of administration

Another significant obstacle is the lack of standardized therapeutic regimens. In infectious disease studies, variables such as MSC dose, administration frequency, delivery route, and treatment timing vary considerably, complicating direct comparisons between studies (148). Experimental evidence indicates that repeated MSC administration may yield greater therapeutic benefits than single-dose treatments. For instance, repeated infusion of genetically engineered Wharton’s jelly-derived MSCs expressing an LL-37 analogue significantly improved antibacterial efficacy and reduced mortality in experimental S. aureus pneumonia without inducing toxicity or immunogenicity (149). However, clinical studies in infectious diseases like COVID-19 have employed diverse dosing strategies, ranging from single low-dose administrations to repeated high-dose infusions (80, 126, 150). Future clinical development must, therefore, include pathogen-specific dose optimization supported by standardized pharmacodynamic and potency biomarkers. A practical standardization approach should combine dose normalization (e.g., cells/kg where appropriate) with predefined product potency criteria and disease-stage biomarkers, rather than relying solely on cell number. Pharmacokinetic considerations for living-cell products should focus on biodistribution, persistence, and exposure within the target inflammatory compartment, while pharmacodynamic assessments should evaluate immune modulation, antimicrobial activity, and tissue repair responses. Biomarker-guided dose escalation could identify the minimal biologically active dose and determine when repeat administration is necessary. Adaptive trial designs may be particularly advantageous in heterogeneous infectious syndromes, allowing dose, interval, or route modifications based on prespecified safety and response criteria while maintaining rigorous statistical control. Such strategies could facilitate indication-specific dosing frameworks rather than a one-size-fits-all MSC dose.

### 5.4 Improving preclinical models

Translation of MSC therapies is further complicated by the limitations of conventional animal models. Although rodent and large-animal models remain indispensable for evaluating safety and biological mechanisms, species-specific differences in immune responses, pathogen susceptibility, and tissue regeneration frequently limit extrapolation to human disease (148, 151). These discrepancies partly explain why promising experimental results are not always reproduced in clinical trials. Advanced human *in vitro* platforms, including organoids and organ-on-chip systems, provide promising complementary models that more faithfully reproduce human tissue architecture, immune responses, and host–pathogen interactions (152, 153). Integration of these platforms into preclinical development may substantially improve prediction of MSC efficacy and facilitate optimization of pathogen-specific therapeutic strategies before clinical evaluation.

### 5.5 Engineering the next generation of MSC therapies

Recent advances suggest that many of the current limitations may be overcome through rational engineering of MSC products. Preconditioning with inflammatory cytokines, hypoxia, vitamin D, or pathogen-associated molecular patterns can enhance antimicrobial peptide production and immunomodulatory activity. Likewise, genetic engineering enables expression of antimicrobial peptides, cytokines, antiviral receptors, or therapeutic proteins that substantially increase antimicrobial efficacy. Biomaterial-based delivery systems and extracellular vesicle therapies further offer opportunities to improve cell survival, tissue retention, and targeted delivery while reducing the risks associated with living cell transplantation.

### 5.6 Future priorities for clinical translation

Collectively, current evidence suggests that the future success of MSC-based anti-infective therapies will depend less on demonstrating efficacy than on improving reproducibility. Four priorities appear particularly important: (i) establishment of standardized manufacturing and quality-control procedures; (ii) development of pathogen-specific potency assays capable of predicting antimicrobial efficacy; (iii) optimization of dosing, timing, and delivery strategies according to disease context; and (iv) advancement of engineered MSCs and cell-free products with enhanced antimicrobial activity. Addressing these challenges will be essential to transform MSCs from promising experimental therapies into reliable and widely applicable clinical interventions for infectious diseases.

## 6 Concluding remarks and future directions

Over the past decade, MSCs have emerged as promising therapeutic candidates for infectious diseases due to their unique ability to integrate direct antimicrobial activity, immune regulation, and tissue regeneration. Unlike conventional antimicrobial therapies that primarily target pathogens, MSCs act at multiple levels of host defense by enhancing pathogen clearance, modulating dysregulated immune responses, limiting tissue damage, and promoting tissue repair. This multifaceted mode of action has generated increasing interest in MSC-based therapies for bacterial, viral, fungal, and parasitic infections, particularly in the context of escalating antimicrobial resistance. A major finding emerging from the current literature is that MSC responses are highly pathogen dependent. Bacterial infections consistently demonstrate the strongest therapeutic benefit, likely because MSCs simultaneously enhance innate antimicrobial defenses and support tissue recovery. In contrast, viral infections reveal a more complex picture in which MSCs primarily mitigate immunopathology rather than directly suppress viral replication. Fungal and parasitic diseases highlight an additional level of complexity, as certain pathogens can exploit MSCs as protective niches or actively reprogram their biological functions. These observations collectively challenge the notion of MSCs as universally beneficial anti-infective agents and instead support a pathogen-specific framework for their therapeutic application. Despite encouraging preclinical and early clinical results, significant barriers remain before MSC-based anti-infective therapies can achieve widespread clinical implementation. MSC heterogeneity, donor variability, tissue source selection, manufacturing processes, dosing regimens, and administration routes continue to contribute to inconsistent outcomes across studies. Furthermore, the lack of standardized potency assays and harmonized reporting criteria limits comparability between investigations and complicates the identification of optimal therapeutic protocols. Future efforts should therefore prioritize the establishment of standardized manufacturing procedures, pathogen-oriented potency testing, and evidence-based guidelines adapted to specific infectious diseases rather than pursuing a universal MSC therapeutic model. An equally important challenge concerns the incomplete understanding of MSC–pathogen interactions. While substantial progress has been made in characterizing MSC immunomodulatory and regenerative functions, the molecular mechanisms governing bidirectional communication between MSCs and pathogens remain poorly understood. This is particularly evident in fungal and parasitic diseases, where pathogens may alter MSC phenotype, survival, differentiation, or immunological activity. Elucidating these interactions will be critical for improving both efficacy and safety.

Another emerging research priority is the regulation of MSC-derived AMPs. Although LL-37, BD2, HAMP, LCN2, and other antimicrobial mediators have been identified as important contributors to pathogen control, the genetic, epigenetic, and metabolic pathways regulating their production remain largely unexplored. Improved understanding of AMP regulation may enable the development of engineered MSCs or cell-free products with enhanced antimicrobial potency. In this context, the establishment of a comprehensive MSC antimicrobial peptide atlas linking AMP expression profiles to microbial targets, mechanisms of action, and therapeutic outcomes could provide a valuable resource for both basic and translational research and accelerate the development of next-generation anti-infective therapies. Looking forward, advances in MSC engineering, preconditioning strategies, extracellular vesicle-based therapeutics, and synthetic biology approaches are likely to expand the therapeutic landscape beyond conventional cell therapy. In parallel, the development of physiologically relevant human organoid and organ-on-chip infection models offers an opportunity to overcome some of the translational limitations associated with animal studies, providing platforms that more accurately recapitulate human host–pathogen interactions and facilitate evaluation of MSC potency and mechanism of action.

In conclusion, MSCs represent a promising platform for future anti-infective therapies, not because they function as conventional antimicrobial agents, but because they simultaneously target infection, inflammation, and tissue injury. The future of the field will likely depend on moving beyond the concept of a universal MSC therapy toward pathogen-tailored cellular and cell-free products designed to address the specific biological challenges posed by different infectious diseases. Such an approach may ultimately position MSC-based therapies as an important component of the next generation of strategies to combat antimicrobial resistance and infection-associated tissue damage.

## References

[1] Yang R Gao H Chen L Fang N Chen H Song G . Effect of peripheral blood-derived mesenchymal stem cells on macrophage polarization and Th17/Treg balance *in vitro*. Regener Ther. (2020) 14:275–83. doi: 10.1016/j.reth.2020.03.008 32455158 PMC7232039

[2] Bhat S Viswanathan P Chandanala S Prasanna SJ Seetharam RN . Expansion and characterization of bone marrow derived human mesenchymal stromal cells in serum-free conditions. Sci Rep. (2021) 11:3403. doi: 10.1038/s41598-021-83088-1 33564114 PMC7873235

[3] Steens J Klar L Hansel C Slama A Hager T Jendrossek V . The vascular nature of lung-resident mesenchymal stem cells. Stem Cells Transl Med. (2021) 10:128–43. doi: 10.1002/sctm.20-0191 32830458 PMC7780817

[4] Ji G Zhang Z Wang X Guo Q Zhang E Li C . Comprehensive evaluation of the mechanism of human adipose mesenchymal stem cells ameliorating liver fibrosis by transcriptomics and metabolomics analysis. Sci Rep. (2024) 14:20035. doi: 10.1038/s41598-024-70281-1 39198546 PMC11358327

[5] Huang J Deng Q Tsang LL Chang G Guo J Ruan YC . Mesenchymal stem cells from perinatal tissues promote diabetic wound healing via PI3K/AKT activation. Stem Cell Res Ther. (2025) 16:59. doi: 10.1186/s13287-025-04141-8 39923118 PMC11807333

[6] Vizoso FJ Costa LA Eiro N . New era of mesenchymal stem cell-based medicine: basis, challenges and prospects. Rev Clínica Esp Engl Ed. (2023) 223:619–28. doi: 10.1016/j.rceng.2023.11.002 38000623

[7] Muthu S Jeyaraman M Kotner MB Jeyaraman N Rajendran RL Sharma S . Evolution of mesenchymal stem cell therapy as an advanced therapeutic medicinal product (ATMP)—An Indian perspective. Bioengineering. (2022) 9:111. doi: 10.3390/bioengineering9030111 35324800 PMC8945480

[8] Alcayaga-Miranda F Cuenca J Khoury M . Antimicrobial activity of mesenchymal stem cells: Current status and new perspectives of antimicrobial peptide-based therapies. Front Immunol. (2017) 8. doi: 10.3389/fimmu.2017.00339 28424688 PMC5371613

[9] Sharma A Chakraborty A Jaganathan BG . Review of the potential of mesenchymal stem cells for the treatment of infectious diseases. World J Stem Cells. (2021) 13:568–93. doi: 10.4252/wjsc.v13.i6.568 34249228 PMC8246252

[10] Murray CJL Ikuta KS Sharara F Swetschinski L Robles Aguilar G Gray A . Global burden of bacterial antimicrobial resistance in 2019: A systematic analysis. Lancet. (2022) 399:629–55. doi: 10.1016/s0140-6736(21)02724-0 35065702 PMC8841637

[11] Bertagnolio S Dobreva Z Centner CM Olaru ID Donà D Burzo S . WHO global research priorities for antimicrobial resistance in human health. Lancet Microbe. (2024) 5:100902. doi: 10.1016/S2666-5247(24)00134-4 39146948 PMC11543637

[12] Shaw TD Krasnodembskaya AD Schroeder GN Zumla A Maeurer M O’Kane CM . Mesenchymal stromal cells: an antimicrobial and host-directed therapy for complex infectious diseases. Clin Microbiol Rev. (2021) 34:e00064-21. doi: 10.1128/CMR.00064-21 34612662 PMC8510528

[13] Silva-Carvalho AÉ Cardoso MH Alencar-Silva T Bogéa GMR Carvalho JL Franco OL . Dissecting the relationship between antimicrobial peptides and mesenchymal stem cells. Pharmacol Ther. (2022) 233:108021. doi: 10.1016/j.pharmthera.2021.108021 34637839

[14] Dürr UHN Sudheendra US Ramamoorthy A . LL-37, the only human member of the cathelicidin family of antimicrobial peptides. Biochim Biophys Acta BBA Biomembr. (2006) 1758:1408–25. doi: 10.1016/j.bbamem.2006.03.030 16716248

[15] Svensson D Nilsson B-O . Human antimicrobial/host defense peptide LL-37 may prevent the spread of a local infection through multiple mechanisms: an update. Inflammation Res. (2025) 74:36. doi: 10.1007/s00011-025-02005-8 40063262 PMC11893641

[16] Simonetti O Cirioni O Goteri G Lucarini G Kamysz E Kamysz W . Efficacy of cathelicidin LL-37 in an MRSA wound infection mouse model. Antibiotics. (2021) 10:1210. doi: 10.3390/antibiotics10101210 34680791 PMC8532939

[17] Verma C Seebah S Low SM Zhou L Liu SP Li J . Defensins: antimicrobial peptides for therapeutic development. Biotechnol J. (2007) 2:1353–9. doi: 10.1002/biot.200700148 17886240

[18] Sung DK Chang YS Sung SI Yoo HS Ahn SY Park WS . Antibacterial effect of mesenchymal stem cells against *Escherichia coli* is mediated by secretion of beta‐ defensin‐ 2 via toll‐ like receptor 4 signalling. Cell Microbiol. (2016) 18:424–36. doi: 10.1111/cmi.12522 26350435 PMC5057339

[19] Johnson EE Wessling-Resnick M . Iron metabolism and the innate immune response to infection. Microbes Infect. (2012) 14:207–16. doi: 10.1016/j.micinf.2011.10.001 22033148 PMC3270215

[20] Navidifar T Meftah E Baghsheikhi H Kazemzadeh K Karimi H Rezaei N . Dual role of hepcidin in response to pathogens. Microb Pathog. (2025) 203:107496. doi: 10.1016/j.micpath.2025.107496 40118299

[21] Jaberi SA Cohen A D’Souza C Abdulrazzaq YM Ojha S Bastaki S . Lipocalin-2: Structure, function, distribution and role in metabolic disorders. BioMed Pharmacother. (2021) 142:112002. doi: 10.1016/j.biopha.2021.112002 34463264

[22] Gupta N Krasnodembskaya A Kapetanaki M Mouded M Tan X Serikov V . Mesenchymal stem cells enhance survival and bacterial clearance in murine *Escherichia coli* pneumonia. Thorax. (2012) 67:533–9. doi: 10.1136/thoraxjnl-2011-201176 22250097 PMC3358432

[23] Harman RM Yang S He MK Van De Walle GR . Antimicrobial peptides secreted by equine mesenchymal stromal cells inhibit the growth of bacteria commonly found in skin wounds. Stem Cell Res Ther. (2017) 8:157. doi: 10.1186/s13287-017-0610-6 28676123 PMC5496374

[24] Bulut Ö Gürsel İ . Mesenchymal stem cell derived extracellular vesicles: Promising immunomodulators against autoimmune, autoinflammatory disorders and SARS-CoV-2 infection. Turk J Biol. (2020) 44:273–82. doi: 10.3906/biy-2002-79 32595362 PMC7314505

[25] Yudintceva N Mikhailova N Fedorov V Samochernych K Vinogradova T Muraviov A . Mesenchymal stem cells and MSCs-derived extracellular vesicles in infectious diseases: from basic research to clinical practice. Bioengineering. (2022) 9:662. doi: 10.3390/bioengineering9110662 36354573 PMC9687734

[26] Gholampour MA Abroun S Nieuwland R Mowla SJ Soudi S . Mesenchymal stem cell‐derived extracellular vesicles conditionally ameliorate bone marrow failure symptoms in an immune‐mediated aplastic anemia mouse model. J Cell Physiol. (2021) 236:6055–67. doi: 10.1002/jcp.30291 33492726

[27] Wang J Huang R Xu Q Zheng G Qiu G Ge M . Mesenchymal stem cell–derived extracellular vesicles alleviate acute lung injury via transfer of miR-27a-3p*. Crit Care Med. (2020) 48:e599–610. doi: 10.1097/CCM.0000000000004315 32317602

[28] Gu X Li Y Chen K Wang X Wang Z Lian H . Exosomes derived from umbilical cord mesenchymal stem cells alleviate viral myocarditis through activating AMPK/mTOR‐mediated autophagy flux pathway. J Cell Mol Med. (2020) 24:7515–30. doi: 10.1111/jcmm.15378 32424968 PMC7339183

[29] Shen Z Kuang S Zhang Y Yang M Qin W Shi X . Chitosan hydrogel incorporated with dental pulp stem cell-derived exosomes alleviates periodontitis in mice via a macrophage-dependent mechanism. Bioact Mater. (2020) 5:1113–26. doi: 10.1016/j.bioactmat.2020.07.002 32743122 PMC7371600

[30] Gao F Chiu SM Motan DAL Zhang Z Chen L Ji H-L . Mesenchymal stem cells and immunomodulation: Current status and future prospects. Cell Death Dis. (2016) 7:e2062–2. doi: 10.1038/cddis.2015.327 26794657 PMC4816164

[31] Song N Scholtemeijer M Shah K . Mesenchymal stem cell immunomodulation: mechanisms and therapeutic potential. Trends Pharmacol Sci. (2020) 41:653–64. doi: 10.1016/j.tips.2020.06.009 32709406 PMC7751844

[32] Wang Y Fang J Liu B Shao C Shi Y . Reciprocal regulation of mesenchymal stem cells and immune responses. Cell Stem Cell. (2022) 29:1515–30. doi: 10.1016/j.stem.2022.10.001 36332569

[33] Li P Ou Q Shi S Shao C . Immunomodulatory properties of mesenchymal stem cells/dental stem cells and their therapeutic applications. Cell Mol Immunol. (2023) 20:558–69. doi: 10.1038/s41423-023-00998-y 36973490 PMC10040934

[34] MacKenzie C Heseler K Muller A Daubener W . Role of indoleamine 2,3-dioxygenase in antimicrobial defence and immuno-regulation: Tryptophan depletion versus production of toxic kynurenines. Curr Drug Metab. (2007) 8:237–44. doi: 10.2174/138920007780362518 17430112

[35] Hui Y Jiao X Yang L Lu D Han Y Yang W . Indoleamine-2,3-dioxygenase: An important controller in maintaining mesenchymal stem cell-mediated immunomodulatory homeostasis. Acta Pharm Sin B. (2025) 15:3404–18. doi: 10.1016/j.apsb.2025.04.022 40698141 PMC12278408

[36] Wang L-T Wang H-H Chiang H-C Huang L-Y Chiu S-K Siu LK . Human placental MSC-secreted IL-1β enhances neutrophil bactericidal functions during hypervirulent Klebsiella infection. Cell Rep. (2020) 32:108188. doi: 10.1016/j.celrep.2020.108188 32997996

[37] Shao Y Wu D Miao C Zhang H . MSC-EVs alleviate sepsis-induced acute lung injury through inhibiting NET-triggered damage to adherens junctions of endothelial cells. Mol Immunol. (2025) 185:6–17. doi: 10.1016/j.molimm.2025.07.004 40664021

[38] Qiu G Zheng G Ge M Huang L Tong H Chen P . Adipose-derived mesenchymal stem cells modulate CD14++CD16+ expression on monocytes from sepsis patients *in vitro* via prostaglandin E2. Stem Cell Res Ther. (2017) 8:97. doi: 10.1186/s13287-017-0546-x 28446249 PMC5406890

[39] Zhang C Liao W Li W Li M Xu X Sun H . Human umbilical cord mesenchymal stem cells derived extracellular vesicles alleviate salpingitis by promoting M1–to–M2 transformation. Front Physiol. (2023) 14:1131701. doi: 10.3389/fphys.2023.1131701 36875046 PMC9977816

[40] Lu Z Chang W Meng S Xu X Xie J Guo F . Mesenchymal stem cells induce dendritic cell immune tolerance via paracrine hepatocyte growth factor to alleviate acute lung injury. Stem Cell Res Ther. (2019) 10:372. doi: 10.1186/s13287-019-1488-2 31801626 PMC6894226

[41] Lu Z Meng S Chang W Fan S Xie J Guo F . Mesenchymal stem cells activate Notch signaling to induce regulatory dendritic cells in LPS-induced acute lung injury. J Transl Med. (2020) 18:241. doi: 10.1186/s12967-020-02410-z 32546185 PMC7298963

[42] Petri RM Hackel A Hahnel K Dumitru CA Bruderek K Flohe SB . Activated tissue-resident mesenchymal stromal cells regulate natural killer cell immune and tissue-regenerative function. Stem Cell Rep. (2017) 9:985–98. doi: 10.1016/j.stemcr.2017.06.020 28781075 PMC5599186

[43] Loh JT Zhang B Teo JKH Lai RC Choo ABH Lam K-P . Mechanism for the attenuation of neutrophil and complement hyperactivity by MSC exosomes. Cytotherapy. (2022) 24:711–9. doi: 10.1016/j.jcyt.2021.12.003 35177337 PMC8843421

[44] Pezzotta A Bovio A Imberti B Locatelli M Corna D Cerullo D . Mesenchymal stromal cell secretome reduces lung injury and thrombo-inflammation induced by SARS-CoV-2 spike protein. Stem Cell Res Ther. (2025) 16:324. doi: 10.1186/s13287-025-04472-6 40597299 PMC12210905

[45] Wang L Deng Z Sun Y Zhao Y Li Y Yang M . The study on the regulation of Th cells by mesenchymal stem cells through the JAK-STAT signaling pathway to protect naturally aged sepsis model rats. Front Immunol. (2022) 13:820685. doi: 10.3389/fimmu.2022.820685 35197984 PMC8858840

[46] Weber MG Walters-Laird CJ Kol A Santos Rocha C Hirao LA Mende A . Gut germinal center regeneration and enhanced antiviral immunity by mesenchymal stem/stromal cells in SIV infection. JCI Insight. (2021) 6:e149033. doi: 10.1172/jci.insight.149033 34014838 PMC8262475

[47] Huang Y Wang J Lai X Qiu Y Cai J Ma Y . Mesenchymal stromal cells attenuate post-stroke infection by preventing caspase-1-dependent splenic marginal zone B cell death. Signal Transduct Target Ther. (2021) 6:60. doi: 10.1038/s41392-020-00415-0 33579897 PMC7881133

[48] Niu X Zhang L Xing S Liu J Li D Wang Y . Targeting lung damage: Amniotic mesenchymal stem cells mitigate lipopolysaccharide-induced acute lung injury via multiple signaling pathways. Int J Mol Sci. (2025) 26:2314. doi: 10.3390/ijms26052314 40076934 PMC11901019

[49] Jeong J Park J-K Shin J Jung I Kim H-W Park A . Inflammatory cytokine-primed MSC-derived extracellular vesicles ameliorate acute lung injury via enhanced immunomodulation and alveolar repair. Stem Cell Res Ther. (2025) 16:450. doi: 10.1186/s13287-025-04576-z 40846969 PMC12374373

[50] Mirshekar M Afkhami H Razavi S Masjedian Jazi F Darban-Sarokhalil D Ohadi E . Potential antibacterial activity and healing effect of topical administration of bone marrow and adipose mesenchymal stem cells encapsulated in collagen-fibrin hydrogel scaffold on full-thickness burn wound infection caused by Pseudomonas aeruginosa. Burns. (2023) 49:1944–57. doi: 10.1016/j.burns.2023.01.005 38042618

[51] Rajesh A Ju EDE Oxford KA Harman RM Van De Walle GR . The mesenchymal stromal cell secretome promotes tissue regeneration and increases macrophage infiltration in acute and methicillin-resistant Staphylococcus aureus-infected skin wounds *in vivo*. Cytotherapy. (2024) 26:1400–10. doi: 10.1016/j.jcyt.2024.06.007 38944795

[52] Kim H Kim O-H Hong H-E Lee S Kim S-J . Harnessing adipose-derived stem cells to release specialized secretome for the treatment of hepatitis B. Int J Mol Med. (2021) 47:15. doi: 10.3892/ijmm.2021.4848 33448314 PMC7834954

[53] Abou Rayia DM Izzularab BM Harras S Ghafar MTA Azzam AR Harras H . Stem cell biotherapy: A new remedy for Trichinella spiralis-induced inflammatory myopathy. Parasitol Int. (2023) 96:102773. doi: 10.1016/j.parint.2023.102773 37330041

[54] Enayati S Halabian R Saffarian P Aghamollaei H Saeedi P . Nisin-preconditioned mesenchymal stem cells combatting nosocomial Pseudomonas infections. Regener Ther. (2024) 26:161–9. doi: 10.1016/j.reth.2024.05.015 38911027 PMC11192785

[55] Yu J Yang M Jin Y Yang K Yang H . Insights into the potential role of BMSCs-exo delivered USP14 on SIRT1 deubiquitination in Staphylococcus aureus-induced model of osteomyelitis. J Orthop Surg. (2025) 20:539. doi: 10.1186/s13018-025-05898-7 40442730 PMC12120999

[56] Li LL Zhu YG Jia XM Liu D Qu JM . Adipose-Derived Mesenchymal Stem Cells Ameliorating Pseudomonas aeruginosa-induced Acute Lung Infection via Inhibition of NLRC4 Inflammasome. Front Cell Infect Microbiol. (2021) 10:581535. doi: 10.3389/fcimb.2020.581535 PMC7820751 33489931

[57] Rangasamy T Ghimire L Jin L Le J Periasamy S Paudel S . Host Defense against Klebsiella pneumoniae Pneumonia Is Augmented by Lung-Derived Mesenchymal Stem Cells. J Immunol. (2021) 207(4):1112–27. doi: 10.4049/jimmunol.2000688 PMC8355100 34341173

[58] Gonzalez H McCarthy S Masterson C . Nebulised mesenchymal stem cell derived extracellular vesicles ameliorate E. coli induced pneumonia in a rodent model. Stem Cell Res Ther (2023) 14:151. doi: 10.1186/s13287-023-03385-6 PMC10245544 37280647

[59] Laterre P-F Sánchez-García M Van Der Poll T De La Rosa O Cadogan K-A Lombardo E . A phase Ib/IIa, randomised, double-blind, multicentre trial to assess the safety and efficacy of expanded Cx611 allogeneic adipose-derived stem cells (eASCs) for the treatment of patients with community-acquired bacterial pneumonia admitted to the intensive care unit. BMC Pulm Med. (2020) 20:309. doi: 10.1186/s12890-020-01324-2 33238991 PMC7686829

[60] Laterre P-F Sánchez García M Van Der Poll T Wittebole X Martínez-Sagasti F Hernandez G . The safety and efficacy of stem cells for the treatment of severe community-acquired bacterial pneumonia: A randomized clinical trial. J Crit Care. (2024) 79:154446. doi: 10.1016/j.jcrc.2023.154446 37918129

[61] Reijnders TDY Laterre P-F François B Sánchez García M Van Engelen TSR Sie D . Effect of mesenchymal stem cells on the host response in severe community-acquired pneumonia. Thorax. (2024) 79:1086–90. doi: 10.1136/thorax-2024-222026 39322407 PMC11503173

[62] Bonfield TL Sutton MT Fletcher DR Folz MA Ragavapuram V Somoza RA . Donor-defined mesenchymal stem cell antimicrobial potency against nontuberculous mycobacterium. Stem Cells Transl Med. (2021) 10:1202–16. doi: 10.1002/sctm.20-0521 33943038 PMC8284776

[63] Yan K Xu G Li Z . MicroRNA-20b carried by mesenchymal stem cell-derived extracellular vesicles protects alveolar epithelial type II cells from Mycobacterium tuberculosis infection *in vitro*. Infect Genet Evol. (2022) 101:105292. doi: 10.1016/j.meegid.2022.105292 35504589

[64] Huang J Wu S Wu M Zeng Q Wang X Wang H . Efficacy of the therapy of 5-aminolevulinic acid photodynamic therapy combined with human umbilical cord mesenchymal stem cells on methicillin-resistant Staphylococcus aureus-infected wound in a diabetic mouse model. Photodiagnosis Photodyn Ther. (2021) 36:102480. doi: 10.1016/j.pdpdt.2021.102480 34375775

[65] Smith AA Bellows CF . Efficacy of paracrine factors from mesenchymal stem cells for treating biofilm-infected wounds in a murine model. J Wound Manag Res. (2022) 18:186–93. doi: 10.22467/jwmr.2022.02040

[66] Khaledi M Ahmadi MH Owlia P Saderi H . Antimicrobial effects of mouse adipose-derived mesenchymal stem cells encapsulated in collagen-fibrin hydrogel scaffolds on Bacteroides fragilis wound infection *in vivo*. Iran BioMed J. (2023) 27:257–68. doi: 10.61186/ibj.27.5.257 37873638 PMC10707812

[67] Ishii R Ohnishi S Hojo M Ishikawa K Funayama E Miura T . Hypoxic culture enhances the antimicrobial activity of amnion-derived mesenchymal stem cells, thereby reducing bacterial load and promoting wound healing in diabetic mice. Biochem Biophys Res Commun. (2024) 739:150903. doi: 10.1016/j.bbrc.2024.150903 39531904

[68] Dubus M Varin J Papa S Rammal H Chevrier J Maisonneuve E . Interaction of Cutibacterium acnes with human bone marrow derived mesenchymal stem cells: A step toward understanding bone implant- associated infection development. Acta Biomater. (2020) 104:124–34. doi: 10.1016/j.actbio.2019.12.028 31881313

[69] Dubus M Varin J Papa S Chevrier J Quilès F Francius G . Bone marrow mesenchymal stem cells offer an immune-privileged niche to Cutibacterium acnes in case of implant-associated osteomyelitis. Acta Biomater. (2022) 137:305–15. doi: 10.1016/j.actbio.2021.10.026 34678484

[70] Khanh VC Fukushige M Chang YH Hoang NN Yamashita T Obata-Yasuoka M . Wharton’s jelly mesenchymal stem cell-derived extracellular vesicles reduce SARS-CoV2-induced inflammatory cytokines under high glucose and uremic toxin conditions. Stem Cells Dev. (2021) 30:758–72. doi: 10.1089/scd.2021.0065 34074129 PMC8356045

[71] Park JH Choi Y Lim C-W Park J-M Yu S-H Kim Y . Potential therapeutic effect of micrornas in extracellular vesicles from mesenchymal stem cells against SARS-CoV-2. Cells. (2021) 10:2393. doi: 10.3390/cells10092393 34572043 PMC8465096

[72] Souza-Moreira L Tan Y Wang Y Wang J-P Salkhordeh M Virgo J . Poly(I:C) enhances mesenchymal stem cell control of myeloid cells from COVID-19 patients. iScience. (2022) 25:104188. doi: 10.1016/j.isci.2022.104188 35402859 PMC8975597

[73] Al Saihati HA Dessouky AA Salim RF Elgohary I El-Sherbiny M Ali FEM . MSC–extracellular vesicle microRNAs target host cell-entry receptors in COVID-19: in silico modeling for *in vivo* validation. Stem Cell Res Ther. (2024) 15:316. doi: 10.1186/s13287-024-03889-9 39304926 PMC11416018

[74] Ebrahim N Al Saihati HA Alali Z Mahmoud SYM Rabaan AA Dessouky AA . Lyophilized MSC-EVs attenuates COVID-19 pathogenesis by regulating the JAK/STAT pathway. Stem Cell Res Ther. (2025) 16:244. doi: 10.1186/s13287-025-04284-8 40369583 PMC12079845

[75] Fathi-Kazerooni M Fattah-Ghazi S Darzi M Makarem J Nasiri R Salahshour F . Safety and efficacy study of allogeneic human menstrual blood stromal cells secretome to treat severe COVID-19 patients: Clinical trial phase I & II. Stem Cell Res Ther. (2022) 13:96. doi: 10.1186/s13287-022-02771-w 35255966 PMC8899458

[76] Zarrabi M Shahrbaf MA Nouri M Shekari F Hosseini S-E Hashemian S-MR . Allogenic mesenchymal stromal cells and their extracellular vesicles in COVID-19 induced ARDS: a randomized controlled trial. Stem Cell Res Ther. (2023) 14:169. doi: 10.1186/s13287-023-03402-8 37365605 PMC10294333

[77] Baghalishahi M Yousefi M Nematollahi-Mahani SN Ziasistani M Pourjafari F Saheli M . Nebulization of mesenchymal stem cells secretome attenuates acute respiratory distress syndrome caused by SARS-CoV-2 corona virus: An open-label, controlled trial. Adv BioMed Res. (2025) 14(1). doi: 10.4103/abr.abr_477_23 40519568 PMC12165292

[78] Gorman EA Rynne J Gardiner HJ Rostron AJ Bannard-Smith J Bentley AM . Repair of acute respiratory distress syndrome in COVID-19 by stromal cells (REALIST-COVID trial): A multicenter, randomized, controlled clinical trial. Am J Respir Crit Care Med. (2023) 208:256–69. doi: 10.1164/rccm.202302-0297OC 37154608

[79] Lightner AL Sengupta V Qian S Ransom JT Suzuki S Park DJ . Bone marrow mesenchymal stem cell-derived extracellular vesicle infusion for the treatment of respiratory failure from COVID-19. CHEST. (2023) 164:1444–53. doi: 10.1016/j.chest.2023.06.024 37356708 PMC10289818

[80] Yuan M-Q Song L Wang Z-R Zhang Z-Y Shi M He J . Long-term outcomes of mesenchymal stem cell therapy in severe COVID-19 patients: 3-year follow-up of a randomized, double-blind, placebo-controlled trial. Stem Cell Res Ther. (2025) 16:94. doi: 10.1186/s13287-025-04148-1 40001244 PMC11863646

[81] Yudhawati R Amin M Rantam FA Prasetya RR Dewantari JR Nastri AM . Bone marrow-derived mesenchymal stem cells attenuate pulmonary inflammation and lung damage caused by highly pathogenic avian influenza A/H5N1 virus in BALB/c mice. BMC Infect Dis. (2020) 20:823. doi: 10.1186/s12879-020-05525-2 33176722 PMC7656227

[82] Zhang M Xu G Zhou X Luo M Ma N Wang X . Mesenchymal stem cells ameliorate H9N2-induced acute lung injury by inhibiting caspase-3-GSDME-mediated pyroptosis of lung alveolar epithelial cells. Eur J Pharmacol. (2023) 960:176148. doi: 10.1016/j.ejphar.2023.176148 37866742

[83] Sun Y Huang S Liu K Tang L Liu X Guo J . Mesenchymal stem cells prevent H7N9 virus infection via rejuvenating immune environment to inhibit immune-overactivity. Biochim Biophys Acta BBA Mol Basis Dis. (2024) 1870:166973. doi: 10.1016/j.bbadis.2023.166973 38029943

[84] Liao Y Fu Z Huang Y Wu S Wang Z Ye S . Interleukin-18-primed human umbilical cord-mesenchymal stem cells achieve superior therapeutic efficacy for severe viral pneumonia via enhancing T-cell immunosuppression. Cell Death Dis. (2023) 14:66. doi: 10.1038/s41419-023-05597-3 36707501 PMC9883134

[85] Anggraeni N Vuong C-K Silvia P Fukushige M Yamashita T Obata-Yasuoka M . Mesenchymal stem cell-derived extracellular vesicles reduce inflammatory responses to SARS-CoV-2 and Influenza viral proteins via miR-146a/NF-κB pathway. Sci Rep. (2024) 14:26649. doi: 10.1038/s41598-024-77258-0 39496662 PMC11535355

[86] Xiao H Yu X Dong Y Bao S Meng X Zhao J . Extracellular vesicles derived from human umbilical cord mesenchymal stem cells alleviated the inflammatory response in mice infected with the influenza virus A (H1N1). Int J Mol Sci. (2025) 26:8839. doi: 10.3390/ijms26188839 41009408 PMC12470093

[87] Qin A Wang X Fu J Shen A Huang X Chen Z . hMSCs treatment attenuates murine herpesvirus-68 (MHV-68) pneumonia through altering innate immune response via ROS/NLRP3 signaling pathway. Mol BioMed. (2023) 4:27. doi: 10.1186/s43556-023-00137-z 37704783 PMC10499773

[88] Chen F Chen Z Wu H-T Chen X-X Zhan P Wei Z-Y . Mesenchymal stem cell-derived exosomes attenuate murine cytomegalovirus-infected pneumonia via NF-κB/NLRP3 signaling pathway. Viruses. (2024) 16:619. doi: 10.3390/v16040619 38675960 PMC11054941

[89] Seong R-K Lee JK Cho GJ Kumar M Shin OS . mRNA and miRNA profiling of Zika virus-infected human umbilical cord mesenchymal stem cells identifies miR-142-5p as an antiviral factor. Emerg Microbes Infect. (2020) 9:2061–75. doi: 10.1080/22221751.2020.1821581 32902370 PMC7534337

[90] Yuan M Tian X Ma W Zhang R Zou X Jin Y . miRNA-431-5p enriched in EVs derived from IFN-β stimulated MSCs potently inhibited ZIKV through CD95 downregulation. Stem Cell Res Ther. (2024) 15:435. doi: 10.1186/s13287-024-04040-4 39563434 PMC11575116

[91] Hashemi ZS Khalili S Barough MS Sarrami Forooshani R Sanati H Sarafrazi Esfandabadi F . Characterization of an engineered ACE2 protein for its improved biological features and its transduction into MSCs: A novel approach to combat COVID-19 infection. Int J Biol Macromol. (2024) 277:134066. doi: 10.1016/j.ijbiomac.2024.134066 39059530

[92] Masalova OV Lesnova EI Klimova RR Momotyuk ED Kozlov VV Ivanova AM . Genetically modified mouse mesenchymal stem cells expressing non-structural proteins of hepatitis C virus induce effective immune response. Vaccines. (2020) 8:62. doi: 10.3390/vaccines8010062 32024236 PMC7158691

[93] Masalova OV Lesnova EI Klimova RR Ivanov AV Kushch AA . Mesenchymal stem cells can both enhance and inhibit the cellular response to DNA immunization by genes of nonstructural proteins of the hepatitis C virus. Int J Mol Sci. (2021) 22:8121. doi: 10.3390/ijms22158121 34360889 PMC8347804

[94] Masalova OV Lesnova EI Kalsin VA Klimova RR Fedorova NE Kozlov VV . Human mesenchymal stem cells modified with the NS5A gene of hepatitis C virus induce a cellular immune response exceeding the response to DNA immunization with this gene. Biology. (2023) 12:792. doi: 10.3390/biology12060792 37372076 PMC10295215

[95] Trujillo-Rodríguez M Viciana P Rivas-Jeremías I Álvarez-Ríos AI Ruiz-García A Espinosa-Ibáñez O . Mesenchymal stromal cells in human immunodeficiency virus-infected patients with discordant immune response: early results of a phase I/II clinical trial. Stem Cells Transl Med. (2021) 10:534–41. doi: 10.1002/sctm.20-0213 33264515 PMC7980217

[96] Wang L Zhang Z Xu R Wang X Shu Z Chen X . Human umbilical cord mesenchymal stem cell transfusion in immune non-responders with AIDS: a multicenter randomized controlled trial. Signal Transduct Target Ther. (2021) 6:217. doi: 10.1038/s41392-021-00607-2 34103473 PMC8187429

[97] Schmidt S Tramsen L Schneider A Schubert R Balan A Degistirici Ö . Impact of human mesenchymal stromal cells on antifungal host response against *Aspergillus fumigatus*. Oncotarget. (2017) 8:95495–503. doi: 10.18632/oncotarget.20753 29221143 PMC5707037

[98] Zhou Y Chen Y Wang S Qin F Wang L . MSCs helped reduce scarring in the cornea after fungal infection when combined with anti-fungal treatment. BMC Ophthalmol. (2019) 19:226. doi: 10.1186/s12886-019-1235-6 31727008 PMC6857224

[99] Trombitaş V-E Nagy AA Berce C Pall E Tăbăran F Ilea A . The role of mesenchymal stem cells in the treatment of a chronic rhinosinusitis—an *in vivo* mouse model. Microorganisms. (2021) 9:1182. doi: 10.3390/microorganisms9061182 34070848 PMC8226609

[100] Bicer M Öztürk E Sener F Hakki SS Fidan Ö . Antifungal efficacy of 3D-cultured palatal mesenchymal stem cells and their secreted factors against *Candida albicans*. ACS Infect Dis. (2025) 11:2894–906. doi: 10.1021/acsinfecdis.5c00657 40973026 PMC12519483

[101] Meng Z Liao Y Peng Z Zhou X Zhou H Nüssler AK . Bone marrow mesenchymal stem-cell-derived exosomes ameliorate deoxynivalenol-induced mice liver damage. Antioxidants. (2023) 12:588. doi: 10.3390/antiox12030588 36978835 PMC10045494

[102] Meng Z Tang M Xu S Zhou X Zhang Z Yang L . Protective effects of bone marrow mesenchymal stem cell-derived exosomes loaded cerium dioxide nanoparticle against deoxynivalenol-induced liver damage. J Nanobiotechnol. (2025) 23:215. doi: 10.1186/s12951-025-03316-y 40098176 PMC11912777

[103] Rodriguez-Echeverri C Puerta-Arias JD González Á . *Paracoccidioides brasiliensis* activates mesenchymal stem cells through TLR2, TLR4, and Dectin-1. Med Mycol. (2021) 59:149–57. doi: 10.1093/mmy/myaa039 32459842

[104] Rodríguez‐Echeverri C Gómez BL González Á . *Histoplasma capsulatum* modulates the immune response, affects proliferation and differentiation, and induces apoptosis of mesenchymal stromal cells. Mycoses. (2023) 66:157–67. doi: 10.1111/myc.13537 36219488

[105] Thakur RS Awasthi V Sanyal A Chatterjee S Rani S Chauhan R . Mesenchymal stem cells protect against malaria pathogenesis by reprogramming erythropoiesis in the bone marrow. Cell Death Discov. (2020) 6:125. doi: 10.1038/s41420-020-00363-2 33298881 PMC7667156

[106] Lima MN Oliveira HA Fagundes PM Estato V Silva AYO Freitas RJRX . Mesenchymal stromal cells protect against vascular damage and depression-like behavior in mice surviving cerebral malaria. Stem Cell Res Ther. (2020) 11:367. doi: 10.1186/s13287-020-01874-6 32843073 PMC7448996

[107] Silva DN Souza BSF Azevedo CM Vasconcelos JF De Jesus PG Feitoza MS . IGF-1-overexpressing mesenchymal stem/stromal cells promote immunomodulatory and proregenerative effects in chronic experimental Chagas disease. Stem Cells Int. (2018) 2018:1–11. doi: 10.1155/2018/9108681 30140292 PMC6081563

[108] Silva DN Souza BSF Vasconcelos JF Azevedo CM Valim CXR Paredes BD . Granulocyte-colony stimulating factor-overexpressing mesenchymal stem cells exhibit enhanced immunomodulatory actions through the recruitment of suppressor cells in experimental Chagas disease cardiomyopathy. Front Immunol. (2018) 9:1449. doi: 10.3389/fimmu.2018.01449 30013550 PMC6036245

[109] Dong L Pu Y Chen X Qi X Zhang L Xu L . hUCMSC-extracellular vesicles downregulated hepatic stellate cell activation and reduced liver injury in S. japonicum-infected mice. Stem Cell Res Ther. (2020) 11:21. doi: 10.1186/s13287-019-1539-8 31918749 PMC6953150

[110] Elnahas N Selim S Rady A Shafey D Sallam HA Taie D . Amelioration of Bilharzial liver fibrosis using mesenchymal stem cells. Egypt J Med Microbiol. (2020) 29:11–9. doi: 10.21608/ejmm.2020.249715

[111] Miranda VHS Gomes TR Eller DE Ferraz LDCN Chaves AT Bicalho KA . Liver damage in schistosomiasis is reduced by adipose tissue-derived stem cell therapy after praziquantel treatment. PloS Negl Trop Dis. (2020) 14:e0008635. doi: 10.1371/journal.pntd.0008635 32853206 PMC7480869

[112] Ellakany AR El Baz H Shoheib ZS Elzallat M Ashour DS Yassen NA . Stem cell-derived exosomes as a potential therapy for schistosomal hepatic fibrosis in experimental animals. Pathog Glob Health. (2024) 118:429–49. doi: 10.1080/20477724.2023.2240085 37519008 PMC11338202

[113] Zanganeh E Soudi S Zavaran Hosseini A Khosrojerdi A . Repeated intravenous injection of adipose tissue derived mesenchymal stem cells enhances Th1 immune responses in Leishmania major-infected BALB/c mice. Immunol Lett. (2019) 216:97–105. doi: 10.1016/j.imlet.2019.10.008 31622634

[114] Zanganeh E Soudi S Zavaran Hosseini A . Intralesional injection of mouse mesenchymal stem cells reduces IL-10 production and parasite burden in L. major infected BALB/c mice. Cell J Yakhteh. (2020) 22(Suppl. 1):11–18. doi: 10.22074/cellj.2020.6838 32779429 PMC7481897

[115] Hamoon Navard S Rezvan H Feiz Haddad MH Baghaban EM Azami S . Expression of cytokine genes in Leishmania major-infected BALB/c mice treated with mesenchymal stem cells. J Med Microbiol Infect Dis. (2020) 8:7–13. doi: 10.29252/JoMMID.8.1.7

[116] Navard SH Rezvan H Haddad MHF Ali SA Nourian A Eslaminejad MB . Therapeutic effects of mesenchymal stem cells on cutaneous leishmaniasis lesions caused by Leishmania major. J Glob Antimicrob Resist. (2020) 23:243–50. doi: 10.1016/j.jgar.2020.09.005 32977079

[117] Koken GY Abamor ES Allahverdiyev A Karaoz E . Wharton jelly derived mesenchymal stem cell’s exosomes demonstrate significant antileishmanial and wound healing effects in combination with aloe-emodin: An *in vitro* study. J Pharm Sci. (2022) 111:3232–42. doi: 10.1016/j.xphs.2022.08.016 35995206

[118] Khodkar I Saki J Arjmand R Saki G Khorsandi L . Adipose-derived stem cells’ secretome attenuates lesion size and parasite loading in leishmaniasis caused by Leishmania major in mice. Iran J Med Sci. (2024) 49:121–129. doi: 10.30476/ijms.2023.96413.2795 38356483 PMC10862109

[119] Ramos TD Silva JD Da Fonseca-Martins AM Da Silveira Pratti JE Firmino-Cruz L Maciel-Oliveira D . Combined therapy with adipose tissue-derived mesenchymal stromal cells and meglumine antimoniate controls lesion development and parasite load in murine cutaneous leishmaniasis caused by Leishmania amazonensis. Stem Cell Res Ther. (2020) 11:374. doi: 10.1186/s13287-020-01889-z 32867857 PMC7457509

[120] Bahrami S Safari M Razi Jalali MH Ghorbanpoor M Tabandeh MR Rezaie A . The potential therapeutic effect of adipose-derived mesenchymal stem cells in the treatment of cutaneous leishmaniasis caused by L. major in BALB/c mice. Exp Parasitol. (2021) 222:108063. doi: 10.1016/j.exppara.2020.108063 33412170

[121] Mashayekh E Khosrojerdi A Hosseini AZ Soudi S . Exploring the impact of Leishmania major on mesenchymal stem cells: Evaluating differentiation, and immunomodulatory function. Iran J Allergy Asthma Immunol. (2024) 22(6):588–99. doi: 10.18502/ijaai.v22i6.14647 38477955

[122] Wang X Mijiti W Jia Q Yi Z Ma J Zhou Z . Exploration of altered miRNA expression and function in MSC-derived extracellular vesicles in response to hydatid antigen stimulation. Front Microbiol. (2024) 15:1381012. doi: 10.3389/fmicb.2024.1381012 38601938 PMC11004373

[123] Wang X Mijiti W Yi Z Jia Q Ma J Xie Z . Immunomodulatory effects of hydatid antigens on mesenchymal stem cells: gene expression alterations and functional consequences. Front Microbiol. (2024) 15:1381401. doi: 10.3389/fmicb.2024.1381401 38655088 PMC11035891

[124] Suresh RV Deng B Gebremicale Y Roche K Miura K Long C . Mesenchymal stem cells of the bone marrow raise infectivity of *Plasmodium falciparum* gametocytes. mBio. (2023) 14:e02232-23. doi: 10.1128/mbio.02232-23 37909740 PMC10746266

[125] Ji Q Liu M Gao L Zhang S Zhang W Wang M . Combination of trimethoprim-sulfamethoxazole and mesenchymal stem cell therapy to treat toxoplasmic encephalitis after hematopoietic stem cell transplantation: A case report. Transpl Immunol. (2024) 87:102130. doi: 10.1016/j.trim.2024.102130 39278332

[126] Leng Z Zhu R Hou W Feng Y Yang Y Han Q . Transplantation of ACE2- mesenchymal stem cells improves the outcome of patients with COVID-19 pneumonia. Aging Dis. (2020) 11:216. doi: 10.14336/AD.2020.0228 32257537 PMC7069465

[127] Gao Y Ji Z Zhao J Gu J . Therapeutic potential of mesenchymal stem cells for fungal infections: Mechanisms, applications, and challenges. Front Microbiol. (2025) 16:1554917. doi: 10.3389/fmicb.2025.1554917 39949625 PMC11821621

[128] Krasnodembskaya A Song Y Fang X Gupta N Serikov V Lee J-W . Antibacterial effect of human mesenchymal stem cells is mediated in part from secretion of the antimicrobial peptide LL-37. Stem Cells. (2010) 28:2229–38. doi: 10.1002/stem.544 20945332 PMC3293245

[129] Yagi H Chen AF Hirsch D Rothenberg AC Tan J Alexander PG . Antimicrobial activity of mesenchymal stem cells against Staphylococcus aureus. Stem Cell Res Ther. (2020) 11:293. doi: 10.1186/s13287-020-01807-3 32680544 PMC7367313

[130] Pezzanite LM Chow L Johnson V Griffenhagen GM Goodrich L Dow S . Toll‐like receptor activation of equine mesenchymal stromal cells to enhance antibacterial activity and immunomodulatory cytokine secretion. Vet Surg. (2021) 50:858–71. doi: 10.1111/vsu.13628 33797775

[131] Krepuska M Mayer B Vitale-Cross L Myneni VD Boyajian MK Németh K . Bone marrow stromal cell-derived hepcidin has antimicrobial and immunomodulatory activities. Sci Rep. (2024) 14:3986. doi: 10.1038/s41598-024-54227-1 38368463 PMC10874407

[132] Saeedi P Halabian R Fooladi AAI . Mesenchymal stem cells preconditioned by staphylococcal enterotoxin B enhance survival and bacterial clearance in murine sepsis model. Cytotherapy. (2019) 21:41–53. doi: 10.1016/j.jcyt.2018.11.002 30477894

[133] Alcayaga-Miranda F Cuenca J Martin A Contreras L Figueroa FE Khoury M . Combination therapy of menstrual derived mesenchymal stem cells and antibiotics ameliorates survival in sepsis. Stem Cell Res Ther. (2015) 6:199. doi: 10.1186/s13287-015-0192-0 26474552 PMC4609164

[134] Cortés-Araya Y Amilon K Rink BE Black G Lisowski Z Donadeu FX . Comparison of antibacterial and immunological properties of mesenchymal stem/stromal cells from equine bone marrow, endometrium, and adipose tissue. Stem Cells Dev. (2018) 27:1518–25. doi: 10.1089/scd.2017.0241 30044182 PMC6209426

[135] Chow L Johnson V Impastato R Coy J Strumpf A Dow S . Antibacterial activity of human mesenchymal stem cells mediated directly by constitutively secreted factors and indirectly by activation of innate immune effector cells. Stem Cells Transl Med. (2020) 9:235–49. doi: 10.1002/sctm.19-0092 31702119 PMC6988770

[136] Marx C Gardner S Harman RM Wagner B Van De Walle GR . Mesenchymal stromal cell-secreted CCL2 promotes antibacterial defense mechanisms through increased antimicrobial peptide expression in keratinocytes. Stem Cells Transl Med. (2021) 10:1666–79. doi: 10.1002/sctm.21-0058 34528765 PMC8641085

[137] Mushahary D Spittler A Kasper C Weber V Charwat V . Isolation, cultivation, and characterization of human mesenchymal stem cells. Cytometry A. (2018) 93:19–31. doi: 10.1002/cyto.a.23242 29072818

[138] Baranovskii DS Klabukov ID Arguchinskaya NV Yakimova AO Kisel AA Yatsenko EM . Adverse events, side effects and complications in mesenchymal stromal cell-based therapies. Stem Cell Investig. (2022) 9:7. doi: 10.21037/sci-2022-025 36393919 PMC9659480

[139] Hazrati A Malekpour K Soudi S Hashemi SM . CRISPR/Cas9-engineered mesenchymal stromal/stem cells and their extracellular vesicles: A new approach to overcoming cell therapy limitations. BioMed Pharmacother. (2022) 156:113943. doi: 10.1016/j.biopha.2022.113943 36411629

[140] Olmedo-Moreno L Aguilera Y Baliña-Sánchez C Martín-Montalvo A Capilla-González V . Heterogeneity of *in vitro* expanded mesenchymal stromal cells and strategies to improve their therapeutic actions. Pharmaceutics. (2022) 14:1112. doi: 10.3390/pharmaceutics14051112 35631698 PMC9146397

[141] Barone PW Wiebe ME Leung JC Hussein ITM Keumurian FJ Bouressa J . Viral contamination in biologic manufacture and implications for emerging therapies. Nat Biotechnol. (2020) 38:563–72. doi: 10.1038/s41587-020-0507-2 32341561

[142] Toppinen M Sajantila A Pratas D Hedman K Perdomo MF . The human bone marrow is host to the DNAs of several viruses. Front Cell Infect Microbiol. (2021) 11:657245. doi: 10.3389/fcimb.2021.657245 33968803 PMC8100435

[143] Cimino M Gonçalves RM Barrias CC Martins MCL . Xeno-free strategies for safe human mesenchymal stem/stromal cell expansion: Supplements and coatings. Stem Cells Int. (2017) 2017:1–13. doi: 10.1155/2017/6597815 29158740 PMC5660800

[144] Han Y Li X Zhang Y Han Y Chang F Ding J . Mesenchymal stem cells for regenerative medicine. Cells. (2019) 8:886. doi: 10.3390/cells8080886 31412678 PMC6721852

[145] Weiss ARR Dahlke MH . Immunomodulation by mesenchymal stem cells (MSCs): mechanisms of action of living, apoptotic, and dead MSCs. Front Immunol. (2019) 10:1191. doi: 10.3389/fimmu.2019.01191 31214172 PMC6557979

[146] Karnieli O Friedner OM Allickson JG Zhang N Jung S Fiorentini D . A consensus introduction to serum replacements and serum-free media for cellular therapies. Cytotherapy. (2017) 19:155–69. doi: 10.1016/j.jcyt.2016.11.011 28017599

[147] Kirsch M Rach J Handke W Seltsam A Pepelanova I Strauß S . Comparative analysis of mesenchymal stem cell cultivation in fetal calf serum, human serum, and platelet lysate in 2D and 3D systems. Front Bioeng Biotechnol. (2021) 8:598389. doi: 10.3389/fbioe.2020.598389 33520956 PMC7844400

[148] Masterson CH Ceccato A Artigas A Dos Santos C Rocco PR Rolandsson Enes S . Mesenchymal stem/stromal cell-based therapies for severe viral pneumonia: Therapeutic potential and challenges. Intensive Care Med Exp. (2021) 9:61. doi: 10.1186/s40635-021-00424-5 34970706 PMC8718182

[149] Gasanov VAO Kashirskikh DA Khotina VA Kuzmina DM Nikitochkina SY Mukhina IV . Preclinical evaluation of the safety, toxicity and efficacy of genetically modified Wharton’s jelly mesenchymal stem/stromal cells expressing the antimicrobial peptide SE-33. Cells. (2025) 14:341. doi: 10.3390/cells14050341 40072070 PMC11898551

[150] Meng F Xu R Wang S Xu Z Zhang C Li Y . Human umbilical cord-derived mesenchymal stem cell therapy in patients with COVID-19: A phase 1 clinical trial. Signal Transduct Target Ther. (2020) 5:172. doi: 10.1038/s41392-020-00286-5 32855385 PMC7450163

[151] Kim H Na DL Lee NK Kim AR Lee S Jang H . Intrathecal injection in a rat model: A potential route to deliver human Wharton’s jelly-derived mesenchymal stem cells into the brain. Int J Mol Sci. (2020) 21:1272. doi: 10.3390/ijms21041272 32070050 PMC7072951

[152] Ahmed Al-Anazi K Al-Anazi KW Al-Jasser MA . The rising role of mesenchymal stem cells in the treatment of various infectious complications. In: Ahmed Al-Anazi K , editor. Update on Mesenchymal and Induced Pluripotent Stem Cells. IntechOpen (2020). doi: 10.5772/intechopen.91475

[153] Kim J Koo B-K Knoblich JA . Human organoids: model systems for human biology and medicine. Nat Rev Mol Cell Biol. (2020) 21:571–84. doi: 10.1038/s41580-020-0259-3 32636524 PMC7339799

